# The Sentinel Phenotype: a theoretical bioenergetic and neurobiological framework for high-fidelity predictive systems (HEPOE Theory)

**DOI:** 10.3389/fnins.2026.1785088

**Published:** 2026-05-29

**Authors:** Camila Leão de Matos Brezolin, Sidnei Brezolin de Freitas

**Affiliations:** Independent Researcher, Brasília, Brazil

**Keywords:** allostatic load, bioenergetics, cognitive biophysics, giftedness, HEPOE Theory, Sentinel Phenotype, predictive coding, Predictive Moral Injury

## Abstract

The HEPOE Theory (High Entropy Predictive Organization Efficiency) proposes a novel conceptual framework for understanding Giftedness (HA/G), moving beyond academic performance-based models toward a biophysical and neuroscientific foundation. Through a theoretical synthesis grounded in the Free Energy Principle and Biological Thermodynamics, the gifted individual is redefined as a “Sentinel”: a high-fidelity sampling system specialized in the early detection of isomorphy and the reduction of systemic entropy. This framework reinterprets Charles Spearman’s general intelligence (*g*) as a macroscopic manifestation of hardware efficiency, where reasoning ability is proposed to be fundamentally constrained by working-memory capacity and the metabolic economy of ATP resynthesis. We hypothesize that the hardware operates under an “open sensory gating” regime and low latent inhibition, leading to high metabolic costs and chronic allostatic load. The paper introduces the original concept of Predictive Moral Injury to conceptualize the potential somatic damage resulting from the early perception of ethical-systemic collapses within low-resolution environments. The HEPOE Unification Matrix integrates decades of classical literature and proposes a rigorous differential diagnosis against the pathologization of ASD, ADHD, and PTSD. It hypothesizes that the Sentinel’s exhaustion is not a dysfunction, but a logistical byproduct of high predictive performance under entropy-saturated conditions.

## 1 Introduction

### 1.1 The blind spot in neuroscience

The study of High Ability/Giftedness (HA/G) often relies on phenomenological descriptions and psychometric metrics that do not fully capture the biological causality of the phenomenon. Confined to terms such as “Giftedness” or “Genius,” these classical nomenclatures emphasize psychometric limits while obscuring the underlying mechanics of the divergent brain and the subject’s biophysical infrastructure. Consequently, they limit validation to only performance that meets institutional expectations. Furthermore, the prefix “Super” suggests a hierarchy of value, whereas this framework posits a biological specialization.

The HEPOE Theory (High Entropy Predictive Organization Efficiency) proposes a break from traditional phenomenological models, such as the Three-Ring Conception (Renzulli, 2005) or the DMGT Model (Gagné, 2004). It postulates that giftedness is not a qualitative “gift” or a purely behavioral phenotype, but a neurobiological hardware configuration specialized in the minimization of free energy (Friston, 2010) in environments of high informational entropy (Shannon, 1948).

Therefore, the present investigation introduces the HEPOE Theory as a novel synthesis between Biological Thermodynamics, Predictive Neuroscience, and Bioenergetics. This framework models giftedness as an optimization function of predictive hardware, linking the metabolic cost of Adenosine Triphosphate (ATP) resynthesis to high-fidelity efficiency in organizing entropic systems.

The fundamental transition between the classical phenomenological view and the new biophysical approach proposed by the theory is summarized in Table 1, which details the shift in focus from behavior to hardware.

**Table 1:** Novel conceptual framework: from behavior to hardware.

Analysis dimension	Classical model (gifted)	HEPOE Model (Sentinel)
Nature of the phenomenon	Phenomenological (gift/talent)	Biophysical (hardware configuration)
Input mechanism	General intelligence/Spearman’s *g* (Spearman, 2008)/(IQ)	Low latent inhibition/Open sensory gating
Processing	Academic/Cognitive performance	Working memory capacity/Free energy minimization
Biological cost	Not measured (abstract)	High metabolic cost (ATP resynthesis)
Hypersensitivity	Personality trait/Intensity	Allostatic load/Systemic vigilance
Data integration	Multidisciplinarity	Universal link (isomorphy and gestalt)
System objective	Success/Personal achievement	Free energy minimization/Homeostasis

Source: Brezolin and Freitas (2026), based on the Free Energy Principle (Friston, 2010) and Information Theory (Shannon, 1948).

Furthermore, the originality of HEPOE lies in its capacity for inclusion. By defining high-fidelity as a hardware configuration, the theory proposes that the Sentinel Phenotype is a biological occurrence that manifests independently of socioeconomic status or cultural background. This explains why “Sentinels” exist in any socioeconomic context, given their hallmark resides not in conventional academic success, but in the intrinsic way their nervous systems organize environmental uncertainty.

### 1.2 Nominal definition: the HEPOE acronym

The HEPOE Theory (High Entropy Predictive Organization Efficiency) defines the biological hardware’s operational flow. The nomenclature follows systems engineering logic:

- High entropy (input): represents the environmental condition of maximum uncertainty (chaos) and the massive sampling rate required by the Sentinel hardware.
- Predictive (process): the system’s operational unit. It replaces reactive behavior with anticipation, utilizing analogies and associations to detect isomorphies within the noise (Bar, 2007).
- Organization (output): the biological objective. It represents the conversion of external entropy into internal logical order, where conceptual metaphors serve as tools for systemic stability (Lakoff and Johnson, 1980).
- Efficiency (constraint): the bioenergetic ratio. It establishes that the metabolic cost of ATP resynthesis strictly limits high-fidelity processing (Bioenergetic Solvency).

### 1.3 The knowledge gap and research objective

Although neuroscience has advanced in mapping the “Connectome,” there remains a critical gap in understanding why certain individuals present systemic exhaustion (burnout) despite high-fidelity predictive performance. Current literature treats high-fidelity sampling and sensory hypersensitivity as distinct phenomena. This study aims to fill this gap by proposing that these are two sides of the same bioenergetic coin. Our objective is to propose the HEPOE Theory as a predictive model for identifying the Sentinel Phenotype, shifting the diagnostic focus from psychometric output to metabolic and informational constraints.

### 1.4 Document structure

This paper is organized into eleven sections. Following this Introduction, Section 2 establishes the core Hypothesis of the Sentinel Phenotype and its biophysical foundations. Section 3 evaluates the hypothesis, unifying classical literature and proposing a robust Differential Diagnosis Matrix. Section 4 explores the biophysical foundations of neurodivergent signal dynamics, while Section 5 defines Twice-Exceptionality (2e) through the physics of wave interference and architectural conflict. Section 6 details the phenomenology of systemic collapse, focusing on the lactate-glutamate crisis and thermal protection protocols. Section 7 deepens the analysis regarding the cost of high-fidelity processing, introducing the original concept of Predictive Moral Injury, the “Spinning in Place” dynamics, and the mathematical formalism of the Brezolin Solvency Integral. Section 8 details the Methodological Framework, characterizing the study as an Integrative Theoretical Synthesis and establishing empirical pathways for falsifiability. Section 9 offers a discussion on the systemic, clinical, and public policy implications of the theory. Section 10 presents final considerations on thermodynamic modulation strategies and future research directions. Finally, Section 11 presents the Conclusion.

## 2 The hypothesis: the Sentinel Phenotype

### 2.1 Defining the Sentinel Phenotype: a shift from psychometrics to bioenergetics

Historically, high cognitive ability, frequently classified as ‘giftedness’, has been predominantly defined utilizing psychometric paradigms. These classical models rely heavily upon standardized intelligence quotients (IQ) and exceptional performance metrics across specific academic or creative domains (Sternberg, 1985; Spearman, 2008). While these traditional frameworks successfully categorize and measure macroscopic cognitive outputs, they inherently obscure the underlying thermodynamic and neurocomputational costs required to generate such high-fidelity results.

Recent systematic reviews (Kuznetsova et al., 2024) highlight the urgent need for a unified model that integrates the cognitive, psychological, and physiological markers of giftedness. The HEPOE framework bridges this exact gap by introducing the thermodynamic mechanics of neural fidelity.

To address this critical epistemological gap, this manuscript proposes the Sentinel Phenotype. We conceptualize this phenotype not as a rejection of classical superior intellectual performance, but rather as the specific, highly sensitive neurocomputational architecture that makes such high IQ scores biologically possible. Consequently, we theoretically define the Sentinel Phenotype as a biological predictive system characterized by four core operational criteria, all of which are grounded in established empirical neurobiology:

1. Atypically high sensory and neurological reactivity: aligning with the “Hyper-brain, Hyper-body” theoretical framework (Karpinski et al., 2018), which empirically links elevated intelligence to physiological hyper-reactivity, this manifests as an acute intolerance to prediction errors and an open sensory gating architecture characterized by reduced latent inhibition (Carson et al., 2003).
2. Accelerated computational rate and working memory capacity: grounded in the Parieto-Frontal Integration Theory (P-FIT) (Jung and Haier, 2007) and the robust correlation between general intelligence (*g*) and working memory (Colom et al., 2004), the hardware enables rapid internal generative model updating.
3. Differentiated bioenergetic recruitment (ATP depletion): consistent with the Neural Efficiency Hypothesis (Neubauer and Fink, 2009), the system exhibits an elevated basal rate of neural energy consumption when driven by continuous, high-resolution active inference in complex environments.
4. Hyper-central coherence and macroscopic integration: while conditions like Autism Spectrum Disorder (ASD) are characterized by localized processing or Weak Central Coherence (Frith, 1989), and ADHD is marked by stochastic signal dispersion and network instability (Castellanos and Proal, 2012), the Sentinel hardware is driven by global integration. We propose the term *Hyper-Central Coherence* to describe the hardware’s imperative to process high-entropy variables specifically to detect universal patterns and macroscopic isomorphies (Bar, 2007), ensuring that raw data is functionally converted into predictive models rather than generating mere behavioral dysregulation.

Within this proposed theoretical framework, a ‘Sentinel’ represents an individual possessing a nervous system evolutionarily optimized to process high-entropy, complex environments with maximum fidelity. However, as a direct thermodynamic consequence of this hyper-reactivity and high metabolic recruitment, this specific phenotype frequently experiences severe allostatic overload and bioenergetic exhaustion when confined within low-entropy, monotonous, or rigid predictive microenvironments.

The long-standing clinical challenge of “uninterpretable” IQ profiles in gifted and twice-exceptional individuals (Gilman et al., 2026) finds a physical explanation within the HEPOE framework. The extreme discrepancies between high-fidelity cognitive indices and lowered processing speeds are not mere statistical outliers, they are modeled to represent the quantifiable ‘processing tax’ predicted by the Solvency Inequality. When the system prioritizes entropy reduction and predictive precision, the remaining metabolic bandwidth for standardized speed tasks may be diminished, resulting in the non-linear scoring patterns that traditional psychometrics struggle to aggregate.

### 2.2 Biological architecture: open gating mechanism and high-entropy processing

The HEPOE Theory conceptualizes the gifted individual as a Sentinel: a high-fidelity biological system responsible for the early detection of anomalies (Panksepp, 2004).

It is proposed that the “Sentinel’s” brain possesses a configuration of low latent inhibition, allowing the input of a massive volume of raw stimuli.

In this context, reasoning ability is fundamentally linked to working-memory capacity, as established by Kyllonen and Christal (1990), forming the biophysical basis for what is psychometrically known as the g factor. While this characteristic is associated with superior creative achievement (Carson et al., 2003), it imposes a homeostatic challenge: organizing noise into coherent patterns. Thus, organizing entropy means transforming the chaos of raw data into useful and predictable knowledge.

HEPOE models giftedness through neural efficiency (Haier, 2016) and the fact that working memory capacity is a core predictor of general intelligence (Colom et al., 2004), confronted with the high maintenance cost of a high-fidelity predictive radar. This is because, unlike standard neural systems, the Sentinel operates under a critical bioenergetic constraint. Processing high-fidelity signals and maintaining synaptic precision require accelerated resynthesis of Adenosine Triphosphate (ATP), as energy is the fundamental constraint on the biological capacities of neurons (Laughlin, 2001).

Consequently, this framework reinterprets the “excitability” described in the Theory of Positive Disintegration (Dabrowski, 1964) as a mechanism for massive data collection. The hypersensitivity characteristic of giftedness is hypothesized as the result of hardware that does not “shut down,” consuming constant chemical energy to maintain the informational stability of the social group, including the metabolic demand for regulatory sleep and synaptic homeostasis (Tononi, 2009).

Supported by the Parieto-Frontal Integration Theory (P-FIT) (Jung and Haier, 2007) and Network Neuroscience (Barbey, 2018), the ability to associate dispersed data into logical structures (Gestalt) functions as the mechanism for organizing entropic systems. Moreover, the core of HEPOE lies in Efficiency of Organization in High Entropy through the “Universal Link,” defined as the capacity to associate disconnected data into a logical structure to detect isomorphies that less sensitive systems would filter as noise.

The HEPOE framework suggests, therefore, that what traditional clinical practice calls “distraction” is actually the collection of raw data to feed the high-fidelity predictive model. This predictive hardware does not merely seek talent but systemic vigilance. Consequently, ethical suffering and intensity emerge as byproducts of the allostatic load (Sterling and Eyer, 1988) imposed by continuous data processing in a high-performance regime. Similarly, from the HEPOE perspective, what classical literature defines as sensory hypersensitivity represents the inevitable cost of an “open sensory gating” system, which acts as an evolutionary high-fidelity sampling strategy grounded in a configuration of low latent inhibition (Carson et al., 2003).

HEPOE Theory conceptualizes “gating” not as a passive sensory dysfunction, but as a high-fidelity sampling strategy. The hardware abdicates the economy of the sensory filter in favor of the precision of the world model, accepting noise as potential data for isomorphy detection, thus justifying the high metabolic cost of ATP (Laughlin, 2001). This occurs because the Sentinel constantly operates at the limit of Cognitive Load (Sweller, 1988; Paas and Sweller, 2014); by keeping the gating open, the hardware is flooded by an informational load that challenges working memory capacity, transforming the organization effort into a chronic metabolic cost.

The Sentinel perceives more environmental variables (light, sound, micro-expressions) because its hardware was selected not to discard *priors* prematurely. This biophysical perspective contextualizes the complaint of “hypersensitivity.” Rather than an emotional choice, it represents a biological configuration where the data entry gate (Input) is wider, consequently requiring greater processing work (Throughput) to avoid saturation.

Giftedness, therefore, is the manifestation of a biological intelligence system operating in a high-performance predictive regime, where ethical suffering and intensity are subproducts of the aforementioned allostatic load imposed by continuous data processing.

The Sentinel archetype is adopted here to describe the functional role of HEPOE hardware in the human ecosystem. Thus, unlike traditional nomenclatures that emphasize the cognitive “bonus,” the “Sentinel” highlights the burden of vigilance: the biological imperative to process entropy before it converts into systemic chaos. To be a Sentinel is to inhabit the frontier between signal and noise, a position that carries a profound allostatic load (Sterling and Eyer, 1988) and an inherent emotional and developmental burden (Miller, 1981).

### 2.3 The thermodynamic-neuronal algorithm

**Figure 1:**
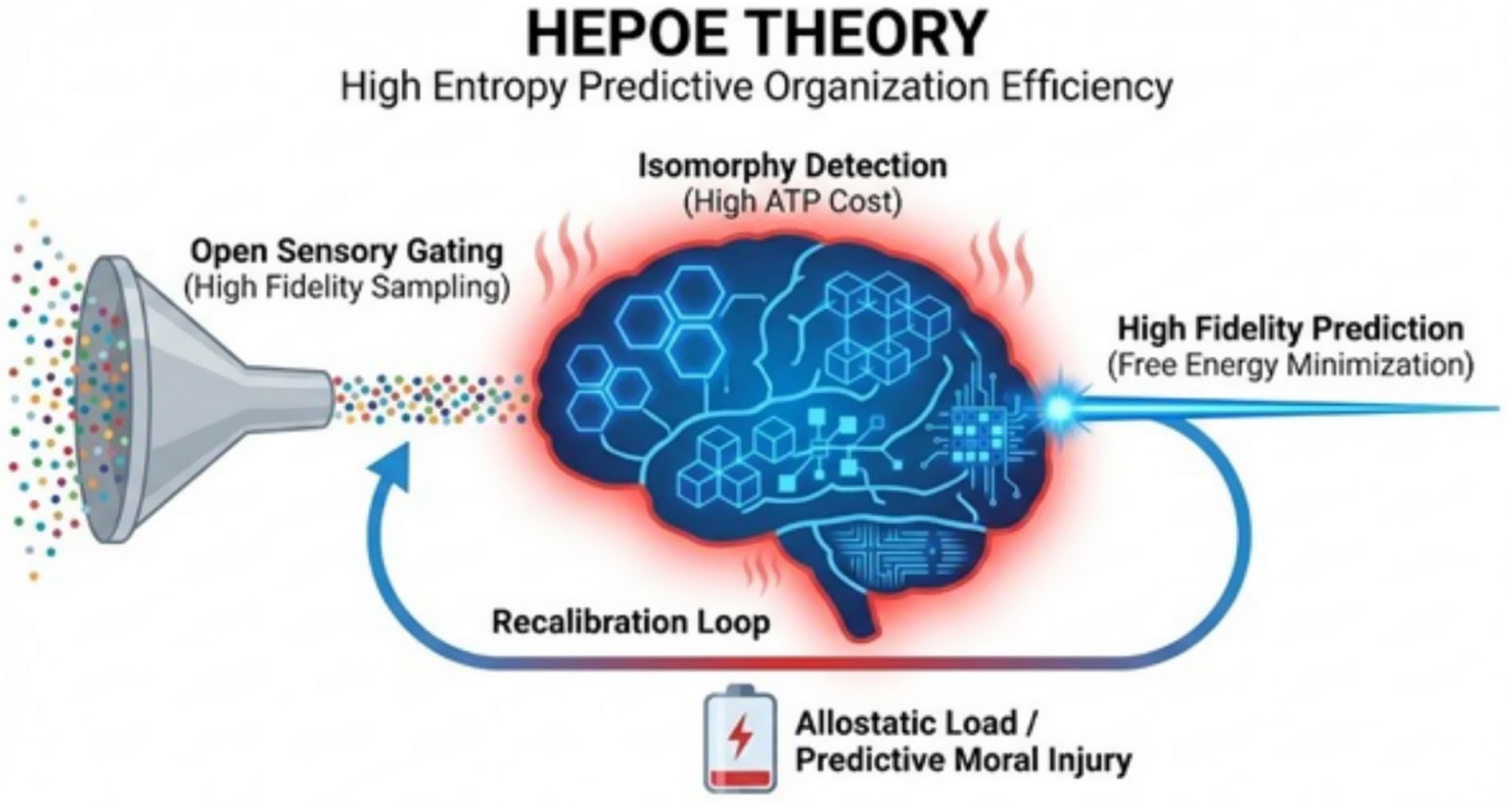
The bioenergetic flow of the Sentinel hardware: from high-entropy input to high-fidelity prediction. The schematic illustrates the transduction of high-entropy input into predictive order. Input: the open sensory gating allows a massive influx of raw data (chaos). Processing: the neural network (Universal Link) organizes data into isomorphies, consuming high levels of ATP (represented by the thermal intensity), reflecting the neuroenergetic cost of parieto-frontal integration (P-FIT) (Jung and Haier, 2007). Output & cost: the system generates a high-fidelity prediction, while the recalibration loop constantly minimizes error. This intensive maintenance results in cumulative Allostatic Load and Predictive Moral Injury when the environment fails to integrate the signal. Source: Brezolin and Freitas (2026).

Three fundamental biophysical variables and one central axiom govern the technical core of the HEPOE framework (see Figure 1):

- High entropy context (H/E): the environment of informational uncertainty that the hardware seeks to organize.
- Predictive processing (P): the operational unit of isomorphy detection that replaces simple reaction with logical anticipation.
- Bioenergetic efficiency (O/E): the critical ratio between predictive accuracy and the metabolic cost of ATP resynthesis.
- The central axiom: the maintenance of synaptic precision requires accelerated ATP resynthesis, identifying energy as the fundamental constraint of biological capacity.

### 2.4 Bioenergetic foundations and neural efficiency

**Figure 2:**
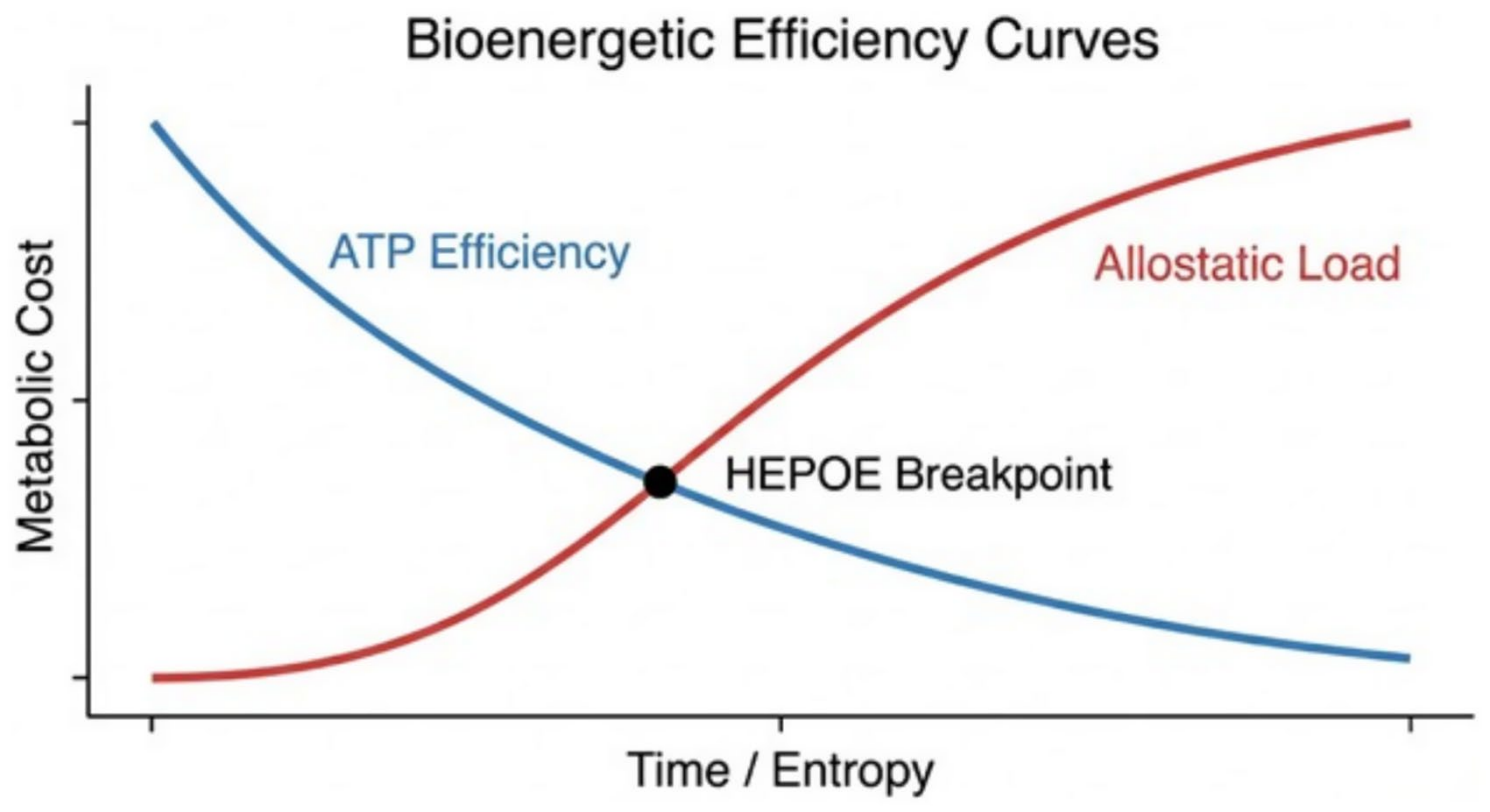
Bioenergetic efficiency curves and the “HEPOE Breakpoint.” The graph correlates the accelerated rate of ATP resynthesis required for synaptic precision with the increment of Allostatic Load over time in high-entropy environments. The “HEPOE Breakpoint” identifies the metabolic collapse (brain fog) when predictive demand exceeds the bioenergetic supply, triggering neuroprotective downregulation. Source: Brezolin and Freitas (2026).

The HEPOE framework postulates that the cognitive capacity of the Sentinel is not merely a psychological trait, but a thermodynamic function constrained by the laws of bioenergetics. Central to this architecture is the premise that high-fidelity predictive processing imposes a substantial metabolic cost, establishing a direct causal link between synaptic precision and energy consumption. Consequently, the Sentinel system does not operate at maximum output indiscriminately; rather, it adheres to a rigorous efficiency protocol (see Figure 2).

The neural efficiency hypothesis (Neubauer and Fink, 2009) governs this protocol, operating through two distinct modalities:

- The resource optimization logic: in low-complexity tasks, the high-fidelity brain exhibits resource economy (glucose/ATP sparing), as the lack of signal-to-noise challenge does not require its specialized hardware, often appearing “disengaged” or lazy. However, it recruits a massive metabolic load specifically to organize high-entropy systems, where high-fidelity sampling is required to identify the Universal Link.
- The dual nature of cognitive shutdown (“brain fog”): based on HEPOE constraints, we classify the phenomenon clinically described as “Brain Fog” into two distinct bioenergetic categories:
  - Type I: metabolic insolvency (depletion): a state of systemic exhaustion resulting from the total consumption of available ATP/Glycogen reserves following prolonged high-fidelity processing. This is equivalent to an engine running out of fuel. It is resolved through rest, nutritional replenishment, and sleep.
  - Type II: adiaphoric throttling (thermal protection): a specific Sentinel reaction to high-entropy environments characterized by systemic incoherence (lies/corruption). Here, reserves may be full, but the hardware initiates a protective downregulation to prevent “thermal runaway” arising from the thermodynamic cost of erasing valid predictions (Landauer’s Principle) (Landauer, 1961) to fit into a low-fidelity environment. This is equivalent to a CPU throttling down to prevent overheating. Rest does not resolve this condition. It requires isomorphic resolution (truth) or removal from the toxic environment.

### 2.5 Mathematical formalism: the HEPOE equation

To formalize the Sentinel Phenotype’s distinct operation, we apply principles from Information Theory and Non-Equilibrium Thermodynamics.

We define the environmental input as a stochastic source *X* with high entropy (*H*), calculated via Shannon’s Entropy formula (Shannon, 1948):


$$H(X)=-\sum p(x)\;\log_{2}\;p(x)$$

Where:

- *H(X)*: the informational entropy of the sensory input [Unit: bits/min].
- *p(x)*: the probability of a given sensory input occurring [Unit: dimensionless].

For neurotypical systems (gated), sensory filters reduce the number of variables before processing. For the Sentinel Phenotype (open gating), the system processes the full spectrum of *H(X)* to find the Universal Link.

We propose that the metabolic cost (*E_met_*) is proportional to the rate of entropy reduction over time. This conversion of informational uncertainty (bits) into biological work (Joules) is non-trivial and fundamentally constrained by thermodynamic limits. Specifically, the lower bound of heat dissipation for information processing (Landauer, 1961), which dictates the minimum energy required to organize or erase neural states.

The HEPOE state (bioenergetic insolvency) occurs when the metabolic demand exceeds the mitochondrial ATP production rate (*ATP_max_*):


$$E_{\mathrm{met}}>\mathrm{AT}P_{\max}$$

Where:

- *E_met_*: the real-time metabolic energy consumption rate required for high-fidelity processing [Unit: *μmol*/g/min].
- *ATP_max_*: the maximum baseline mitochondrial production rate [Unit: *μmol*/g/min].

Under these conditions, the system accumulates Allostatic Load (*L*) (Sterling and Eyer, 1988) over time (*t*), leading to the systemic exhaustion observed clinically:


$$L=\int_{0}^{t}(E_{\mathrm{met}}\;(t)–\mathrm{AT}P_{\max})\mathrm{dt}$$

Where:

- *L*: the cumulative bioenergetic debt (Allostatic Load) [Unit: *μmol*/g].
- *E_met_*: the real-time metabolic energy consumption rate required for high-fidelity processing [Unit: *μmol*/g/min].
- *ATP_max_*: the maximum baseline mitochondrial production rate [Unit: *μmol*/g/min].
- *t*: time of exposure to high-entropy environment [Unit: min].

This integral represents the accumulation of bioenergetic debt, manifesting as “Predictive Moral Injury” when the system fails to minimize environmental surprise due to energetic collapse.

## 3 Evaluation of the hypothesis

### 3.1 Critical literature review (unification of classical literature)

Performance and behavior often serve as the foundations of the most widely accepted theories in educational institutions worldwide. Renzulli (2005) focuses on the intersection of Above-Average Ability, Task Commitment, and Creativity, while Sternberg (1985) highlights the Triarchic Theory of Intelligence, comprising Analytical, Creative, and Practical (real-world success).

The HEPOE approach does not focus on the “product” (the rings or the success), but on the neurobiological process that generates these behaviors.

Other theorists explain how gifts convert into talents. Gagné (2004) demonstrates that this process occurs through environmental and intrapersonal catalysts. Gardner (1983) proposes that intelligence is not a unitary construct but manifests in multiple domains (Musical, Logical-Mathematical, Spatial, Interpersonal, among others).

HEPOE unifies these multiple intelligences under the concept of Entropy Organization: regardless of the domain of activity, the hardware performs the same fundamental operation of pattern extraction and uncertainty reduction with high fidelity.

Furthermore, some studies focus on the internal experience and vulnerability of the individual. Dabrowski (1964) defines Overexcitabilities as the basis for the gifted individual’s intensity, while Terrassier (1979) identifies Dyssynchrony, defined as the gap between precocious intellectual development and affective/social spheres.

HEPOE offers a physical cause for this phenomenon: Dabrowski’s (1964) “overexcitabilities” are restructured as functional consequences of Low Latent Inhibition and High Synaptic Gain; and dyssynchrony is redefined as a processing speed mismatch between distinct hardware resolutions, where the Sentinel’s high-fidelity predictive performance collides with systems (social or biological) of lower informational resolution.

Finally, more recent studies seek the physical substrate of intelligence. Jung and Haier (2007) provide evidence for the efficiency of the network between the Frontal and Parietal lobes, while Neubauer and Fink (2009) highlight Neural Efficiency (glucose economy and resource optimization).

The HEPOE Theory synthesizes these findings into a Predictive Processing framework, proposing that the gifted brain achieves higher efficiency by organizing entropy predictively. The correlation between the pillars of the most accepted theories in the literature and the reinterpretation offered by predictive logic is presented in Table 2.

**Table 2:** Comparison between classical theories and HEPOE.

Aspect	Classical theories	HEPOE Theory
General intelligence	Spearman’s *g* factor (psychometric correlation/mental energy) (Spearman, 2008)	Universal Link (bioenergetic efficiency in isomorphy detection)
Focus	Performance, success, and IQ	Biophysics, predictive processing, and entropy
Cause	Genetic or environmental variability	Evolutionary specialization (Sentinel hardware)
Sensitivity	Overexcitability (behavioral trait)	Low latent inhibition and open gating (mechanism)
Error	Perfectionism (personality trait)	Prediction error minimization (bioenergetic precision vs. ATP cost)
Inclusion	Psychometric and achievement criteria	Systemic Function Phenotyping

Source: Brezolin and Freitas (2026), synthesized and adapted from Spearman (2008), Dabrowski (1964), Renzulli (2005), and Silverman (1993).

This review suggests, therefore, that HEPOE does not invalidate classical constructions but offers the bioenergetic substrate they lacked. By conceptualizing that perfectionism is, in fact, an “Intolerance to Prediction Error” due to the high ATP cost of recalibrating the model, we transition from a descriptive psychology toward a causal neuroscientific perspective.

### 3.2 Phenomenological scope and field unification

The HEPOE framework proposes the biological substrate for Charles Spearman’s g factor (2008). While Spearman identified a universal correlation across diverse cognitive tasks and hypothesized a form of “mental energy,” HEPOE redefines this energy as the metabolic efficiency of ATP resynthesis and the network’s capacity to maintain signal fidelity across the P-FIT architecture (Jung and Haier, 2007). Thus, the general factor g is understood here as the system’s global efficiency in organizing entropy through the Universal Link.

HEPOE Theory proposes the biophysical substrate for phenomena that the classical literature on High Abilities described phenomenologically but lacked a unified causal basis. By defining the Sentinel as a high-fidelity sampling system, HEPOE provides the “hardware” mechanism for:

- The dynamics of intensity and sensitivity: overexcitability and Positive Disintegration (Dabrowski, 1964; Mendaglio, 2008) function as the operational baseline of an open gating hardware. Consequently, this framework redefines hypersensitivity not as a personality trait, but as the inevitable metabolic cost of this open sensory gating system. This high-fidelity sampling strategy mandates a higher throughput, where ‘intensity’ is the phenomenological manifestation of the accelerated ATP resynthesis required to maintain informational stability. This explains Sensory Hypersensitivity and Perfectionism (Pyryt, 2002, 2004) as attempts to regulate a high-fidelity signal.
- Development and leap cognition: asynchrony (Silverman, 1993) and “Leap Learning” result from the efficiency of the Universal Link in processing isomorphies. This utilizes Associative Long-Term Memory and Abstract Thinking that operate under the logic of entropy reduction, challenging the limits of Working Memory (Sweller, 1988).
- Identity and social cost: masking/camouflaging (Hull et al., 2017) and imposter syndrome (Clance and Imes, 1978) serve as output management strategies to mitigate Allostatic Load (Sterling and Eyer, 1988) and Predictive Moral Injury in low-entropy environments. These arise from ethical-systemic mismatch, directly impacting the academic self-concept of the Sentinel (Beckmann et al., 2020). Meanwhile, the Meltdown phenomenon, unlike a “tantrum” (which has social or manipulative intent), is an involuntary physiological response to sensory or cognitive overload, typified by the dysregulation of the autonomic nervous system when internal entropy exceeds the hardware’s processing capacity.
- Cognitive processing: associative long-term memory and abstract thinking are grounded in the continuous search for free energy reduction. This transforms the “shadow side” and sleep disturbances into subproducts of the high ATP cost (Laughlin, 2001).
- Biological and systemic variables: the impact of Gender Perspective (Reis, 2002) and cross-cultural personality variations (Costa et al., 2001) are integrated as variables that modulate the Sentinel’s expression through epigenetic interactions. This facilitates a rigorous Differential Diagnosis between hardware specialization and processing pathologies.

In this way, the translation of the Sentinel hardware into observable behavior manifests through a set of phenomena exhaustively described by classical literature but often treated as isolated traits or personality idiosyncrasies. Under the lens of HEPOE Theory, concepts such as Asynchrony (Silverman, 1993) and Overexcitability (Dabrowski, 1964) cease to be mere phenotypic descriptions and are understood as logistical consequences of a system operating in high sensory resolution.

Perfectionism, for example, is no longer seen merely as a personality trait. Instead, within this framework, it is identified as a technical effort toward precision: the hardware’s attempt to align external reality with its high-fidelity internal predictive model. Similarly, social survival mechanisms, such as Masking and Imposter Syndrome, are revealed as strategies for output management and ATP economy (Laughlin, 2001) in low-complexity environments.

To systematize this integration, Table 3 presents the Systemic and Phenomenological Unification Matrix, linking classical authors to HEPOE’s hardware mechanisms.

**Table 3:** Systemic and phenomenological unification matrix (HEPOE).

Category	Referenced phenomena and authors	Reinterpretation by HEPOE Theory (hardware mechanism)
Capture architecture (input)	Overexcitability (Dabrowski, 1964); Sensory gating (Gere et al., 2009); Reduced latent inhibition (Carson et al., 2003).	Readiness state of hardware with “open” sensory gates; high-fidelity sampling strategy that accepts noise as potential data.
Processing efficiency (throughput)	Processing speed (Jung and Haier, 2007); General intelligence/g factor (Spearman, 2008); Associative/working memories (Bechtereva, 1978; Kyllonen and Christal, 1990).	High performance in the P-FIT network (Jung and Haier, 2007); accelerated search for isomorphies via the Universal Link and free energy reduction for informational stabilization.
Convergence dynamics (gestalt)	Leap learning (Rogers, 1986); Precocious abstract thinking (Hollingworth, 1942); Multiple intelligences (Gardner, 1983); Triarchic intelligence (Sternberg, 1985).	Non-linear processing; innate ability to organize high entropy into complex logical structures and pattern detection where standard systems see only noise.
Overload management and burden	Perfectionism (Pyryt, 2002); Asynchrony (Silverman, 1993); Cognitive load (Sweller, 1988; Paas and Sweller, 2014); Meltdown vs. Tantrum; heredity/longevity (Terman, 1925).	Attempt to reduce prediction error to maintain model precision; logistical mismatch between processing demand and biological infrastructure (ATP/Allostatic Load).
Identity and systemic impact (output)	Social camouflaging (Hull et al., 2017); Imposter syndrome (Clance and Imes, 1978); Predictive Moral Injury (Brezolin and Freitas, 2026); Gender perspective (Reis, 2002).	Data output management for Allostatic Load mitigation and social protection; real somatic injury resulting from early detection of ethical collapses and systemic failures (Sentinel).

Source: Brezolin and Freitas (2026), synthesized and adapted from Dabrowski (1964), Jung and Haier (2007), Spearman (2008), Kyllonen and Christal (1990), and the collective works of the referenced authors.

Upon observing the Unification Matrix, it becomes evident that HEPOE Theory does not discard the observations of Renzulli, Gagné, or Silverman; on the contrary, it provides a biophysical foundation for them.

The integration of concepts such as Predictive Moral Injury and Gender Perspective variables (Reis, 2002) allows the theory to transition from cellular biology to political sociology without losing technical rigor.

This unification is what finally enables the development of a robust Differential Diagnosis. If all these phenomena, ranging from sleep patterns to perfectionism, are subproducts of specialized high-fidelity hardware rather than a defective system, the distinction between the Sentinel and processing pathologies ceases to be subjective and becomes an analysis of signal purpose. In the following chapter, we will explore how this architecture fundamentally distinguishes itself from ASD, ADHD, and PTSD, resolving the ambiguities that have historically led to the pathologization of high-fidelity systems.

Ultimately, HEPOE Theory aims to prevent iatrogenic harm, defined as damage caused by the healthcare system itself or by inaccurate diagnoses that attempt to treat hardware as a signal pathology.

### 3.3 Hardware differential diagnosis: HEPOE vs. ASD, ADHD, and PTSD

Differential diagnosis from the HEPOE perspective is not based on the observation of isolated behaviors, but on the purpose of processing. The Sentinel Phenotype, as proposed by the HEPOE framework, operates as a high-fidelity sampling system that must be distinguished from Autism Spectrum Disorder (ASD), Attention Deficit Hyperactivity Disorder (ADHD), and Post-Traumatic Stress Disorder (PTSD).

In this context, phenomena such as perfectionism, extensively documented by Pyryt (2002, 2004), are no longer viewed as mere personality traits. Through the lens of HEPOE, perfectionism is redefined as an Intolerance to Prediction Error: a technical hardware requirement to ensure signal fidelity and avoid ATP waste in the recalibration of imprecise models. While in ASD rigidity is defensive and in ADHD attention is diffuse, in the Sentinel, rigor is a logistical strategy for processing optimization.

Thus, while the symptomatology may overlap, the hardware architecture reveals distinct systemic objectives and thermodynamic signatures:

- HEPOE vs. ASD (Holistic Tuning vs. High-Precision Singularity): in ASD, literature describes Weak Central Coherence (Frith, 1989), where the system prioritizes local detail over global integration to achieve near-zero entropy within specialized predictive fields. The “rigidity” observed is a thermodynamic defense against unpredictable external noise. In the Sentinel, the opposite phenomenon occurs: Hyper-Central Coherence. The hardware captures detail with high fidelity (Open Gating) exclusively to feed the search for isomorphies and global patterns. Unlike signal fragmentation in ASD, the Sentinel operates in Holistic Tuning, where the detail serves as the necessary input for Gestalt validation.
- HEPOE vs. ADHD (Overload vs. Stochastic Sampling Strategy): ADHD represents a strategy of extensive environmental scanning characterized by a dysregulation in the dopaminergic gain. Predictive energy (ATP) is dispersed across multiple simultaneous high-entropy threads, resulting in a continuous “metabolic leak” toward novel stimuli. In the Sentinel, “distraction” is actually a cognitive load overflow (Sweller, 1988). The hardware does not suffer from a lack of focus, but from an excess of raw data sampling that saturates working memory. Where ADHD presents a failure in maintaining attention due to under-stimulation (seeking dopamine), the Sentinel tends to exhibit exhaustion due to over-stimulation, where “fog” is a protective hardware shutdown to prevent oxidative damage from excessive ATP depletion.
- HEPOE vs. PTSD (Proactive vs. Reactive Vigilance): PTSD manifests as reactive hypervigilance, where the system becomes hypersensitive to specific danger triggers following a traumatic event. Conversely, the Sentinel exhibits an Innate Evolutionary Vigilance that precedes trauma. The ethical suffering and Predictive Moral Injury of the Sentinel do not arise from a past wound, but from the capacity to predict the future collapse of ethical and social systems. It is a proactive and systemic hypervigilance requiring a clinical approach of hardware support rather than mere desensitization.

Understanding the Sentinel as a high-fidelity sampling system allows HEPOE Theory to serve as an integrating axis for the field of High Abilities. Various authors, over decades, have described isolated facets of this hardware, whether through the lens of behavior, creativity, or sensitivity. The Sentinel Phenotype operates at the critical point between these neurodivergent states: it possesses the broad entropy capture of ADHD and the high-resolution processing of ASD, but its unique burden is Allostatic Overload.

**Figure 3:**
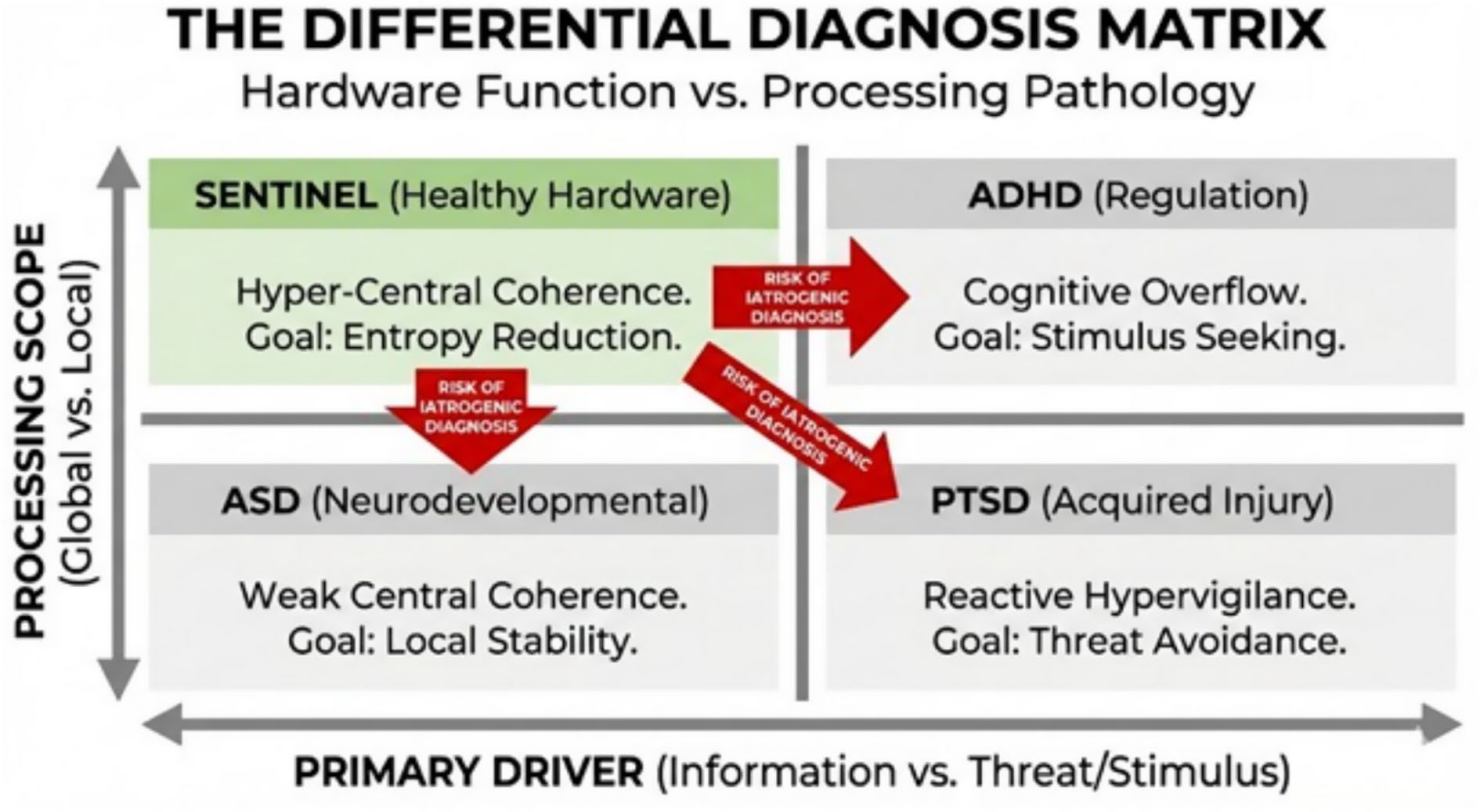
The differential diagnosis matrix. A comparative framework to prevent diagnostic errors. The matrix contrasts the Sentinel’s Hyper-Central Coherence (focused on global Gestalt reduction) against the processing signatures of ASD (Local Stability), ADHD (Stimulus Seeking), and PTSD (Reactive Hypervigilance). Arrows indicate the risk of Diagnostic Iatrogenesis when the Sentinel hardware is treated as a pathology rather than a specialized phenotype. Source: Brezolin and Freitas (2026).

The architectural distinctions between the Sentinel and the processing pathologies most frequently confused in clinical practice are consolidated in Table 4, focusing on the evolutionary purpose of the system (see Figure 3).

**Table 4:** Hardware differential diagnosis (HEPOE vs. Pathologies).

Condition	Hardware mechanism (gating/processing)	System objective (purpose)
Sentinel Phenotype (HEPOE) (Brezolin and Freitas, 2026)	Open high-fidelity gating. Massive sampling and signal processing in high-resolution regime, characterized by high metabolic cost (Laughlin, 2001) and reduced latent inhibition (Carson et al., 2003).	Entropy reduction: Extraction of isomorphies and synthesis of complexity for systemic prediction.
ASD (Autism) (Frith, 1989)	Rigid/Inconsistent gating. Prioritization of local detail; failure in global integration (weak central coherence).	Local stability: System protection through variable reduction and pursuit of immutability.
ADHD [American Psychiatric Association (APA), 2013]	Dopaminergic gain dysfunction. Noise in signal prioritization; instability in sustaining attentional load.	Stimulus seeking: Homeostasis via immediate reward to compensate for low baseline cortical tone.
PTSD (Trauma) (Litz et al., 2009)	Reactive gating (hypervigilance). Gate opening conditioned to memories of fear and specific threats.	Survival: Detection of immediate dangers based on past traumas (Focus on Danger, not Pattern).

Source: Brezolin and Freitas (2026), based on clinical criteria from American Psychiatric Association (APA) (2013) and neurobiological benchmarks from Laughlin (2001) and Carson et al. (2003).

The presented matrix highlights that HEPOE Theory not only encompasses legacy knowledge but also resolves persistent diagnostic ambiguities. By defining the Sentinel’s hardware by its evolutionary purpose (the detection of isomorphies for the reduction of systemic entropy), it becomes evident that what is frequently labeled as pathology (ASD, ADHD, or PTSD) possesses distinct processing signatures. While in processing pathologies the system exhibits rigidity or failures in signal maintenance, in the Sentinel, the challenge is purely logistical: managing the massive volume of raw data and the consequent “Predictive Moral Injury” arising from the anticipation of collapses. Therefore, ethical suffering and exhaustion are not system failures, but the biological cost of its operation in a high-performance predictive regime.

To ensure falsifiability, HEPOE Theory distinguishes itself from purely psychometric IQ models or generalized anxiety disorders. The Sentinel Phenotype is defined by the strong correlation between high-resolution predictive precision and its proportional ATP metabolic cost. Therefore, a system that exhibits high cognitive performance without the specific allostatic load or bioenergetic insolvency markers does not fall under the HEPOE Sentinel definition.

## 4 Biophysical foundations: neurodivergent signal dynamics (ASD, ADHD, and PTSD)

### 4.1 Processing dynamics and signal architectures

While ASD and ADHD are frequently viewed as comorbid diagnoses, from the perspective of neural thermodynamics, they represent distinct signal management strategies. Therefore, to understand Twice-Exceptionality (2e) under the HEPOE Theory, it is necessary to transcend the view of “comorbidity” and analyze neurobiological dynamics in isolation before observing how they interact with the High-Fidelity Structural Substrate (Sentinel).

This framework posits that the Sentinel System is defined by its High-Fidelity, a modern thermodynamic reinterpretation of what Luria (1973) described as Cortical Tone, defined as the optimal state of cortical activation necessary for complex mental processes. While Luria focused on the regulation of this tone by the brainstem and reticular formation, HEPOE defines it as the system’s capacity to sustain high-fidelity information loads without thermal or metabolic collapse. Thus, Twice-Exceptionality is not an overlap of clinical conditions, but the manifestation of a Sentinel System operating under specific signal processing dynamics (ADHD, ASD, or PTSD).

### 4.2 The foundation: the Sentinel system

Each phenotype represents a distinct strategy for handling information flow and minimizing uncertainty. The Sentinel System is defined by a High-Fidelity structural architecture, operating under an intrinsic connectivity pattern oriented toward meta-prediction and the search for isomorphy (Baron-Cohen, 2020). While the structural substrate ensures the lossless integrity of the signal through thalamic amplification (Schmitt et al., 2017), its primary connectivity pattern is programmed for isomorphy extraction. This involves the constant search for global patterns that ensure system solvency in complex environments, aligning with Global Central Coherence (Frith and Happé, 1994), an algorithm that seeks to reduce entropy through the identification of universal patterns.

Unlike the neurotypical brain, which utilizes heuristics to discard “noise,” the Sentinel maintains an open gating to preserve information integrity. This state is sustained by a thalamic amplification of cortical connectivity, which, instead of filtering, enhances signal transmission to the cortex (Schmitt et al., 2017). This constitutes the high-fidelity foundation upon which other dynamics are installed.

Twice-Exceptionality (2e) occurs when this native high-order connectivity pattern is forced to run simultaneously with divergent signal processing subroutines (ASD, ADHD, or PTSD), generating the interference phenomena described hereafter. While the traditional view suggests an additive model of comorbidity, we propose a model grounded in Information Theory (Shannon, 1948).

#### 4.2.1 Fidelity levels

In HEPOE Theory, the gradation of Giftedness can be redefined by the Processing Fidelity and the Phase Synchrony Stability of the Sentinel’s structural substrate:

- Nominal data throughput (P-FIT): the Deep Sentinel may possess networks with superior energy efficiency, allowing massive volumes of data to cross the system with minimal loss of integrity (Haier et al., 1988). The P-FIT “bridge” operates in a high-conductivity regime, facilitating macro-structural synthesis.
- Channel capacity and fidelity (lossless): based on the Mathematical Theory of Communication (Shannon, 1948), the high-fidelity neural architecture keeps the signal “clean” even under high voltage. The Deep Sentinel may possess a system that accurately separates signal from noise, allowing the native connectivity pattern to execute the isomorphy extraction algorithm without data degradation. Consequently, the signal is preserved in its maximum purity before interpretation.
- Phase synchrony and propagation time: profound giftedness requires the Phase Velocity to be constant to avoid signal dispersion. Greater structural integrity may allow the structural substrate to maintain multiple prediction threads in perfect synchrony, minimizing the temporal jitter that would otherwise destabilize scenario prediction (Schmitt et al., 2017).

#### 4.2.2 The variable *H(E)*: total sampling dynamics

The first point of saturation lies in the *H(E)* variable. In typical architectures, the Thalamic Reticular Nucleus (TRN) acts as a Logic Gate for GABAergic lateral inhibition (Crick, 1984; Pinault, 2004), rejecting low-value data. In the 2e phenotype, consistent with the “Leaky Thalamus Hypothesis” (Schmitt et al., 2017), the system operates in Full Sampling mode, serving as the biophysical correlate of Low Latent Inhibition (Carson et al., 2003). Background noise and facial micro-expressions are processed with the same clock priority as the primary task, raising the system’s Noise Floor to critical levels.

#### 4.2.3 The variable Δ*P*: the inference loop

Faced with an uncompressed data stream, the Sentinel algorithm does not ignore chaos; it attempts to decode it. The brain applies High-Order Bayesian Inference to find patterns in stochastic (random) signals. This effort of Free Energy Minimization (Friston, 2010), operating under a High-Fidelity regime, prevents the simplistic compression of raw data, creating an infinite processing loop that rapidly drains available ATP.

This high-order Bayesian inference loop allows the Sentinel system to perform a solvency meta-prediction. Far from a subjective “intuition,” it is a probabilistic calculation based on the prediction error rate versus the availability of metabolic resources (Laughlin, 2001). Under the lens of the Free Energy Principle (Friston, 2010), this monitoring constitutes High-Precision Interoception (Lawson et al., 2014). However, maintaining this high-resolution interoceptive vigilance imposes its own thermodynamic cost, exacerbating the demand for ATP (Laughlin, 2001) and narrowing the solvency margin.

The “knowledge that the collapse is coming” is, therefore, the conscious output of a precision neurobiological monitoring, acting as an Early Warning System (EWS). This system utilizes the high-fidelity infrastructure to identify the prediction error trajectory and the increase in internal entropy. Although the EWS emits the interoceptive alert signal, the system’s inability to reduce the load (due to environmental imperatives or the cost of Masking) leads to final saturation. Thus, the collapse does not occur due to a lack of system warning, but due to maneuver insolvency: the high-fidelity neural architecture detects the imminent impact, but processing rigidity or external demand prevents the execution of mitigation protocols in time.

### 4.3 The ASD dynamic: resonance and local systematization

In isolated ASD, the system, despite having an open sensory gating, operates under a regime of local resonance driven by an imbalance in excitatory/inhibitory (E/I) signaling (Rubenstein and Merzenich, 2003). This results in a high-order precision gain on specific local signals, leading to excessive local synchrony at the expense of global integration (Belmonte et al., 2004). The neural architecture assigns excessive gain (weight) to the precision of specific signals (local signals), ignoring the global context to preserve the integrity of the raw data. It is a “High-Resolution” system within a narrow spectrum. It seeks to reduce uncertainty through extreme systematization.

Energy is, therefore, concentrated in signal depth (depth of detail). The metabolic cost does not stem from dispersion, but from intensity: the system “vibrates” with maximum energy on specific signals (high-order precision), which generates high cognitive inertia. The state transition (task or focus switching) is extremely costly, requiring a massive expenditure of ATP to “deactivate” the previous resonance.

#### 4.3.1 The nature of support levels: a biophysical perspective

The gradation of ASD into “Support Levels” (1, 2, and 3) is frequently interpreted through a purely functional or behavioral lens. However, from the perspective of HEPOE Theory, we propose that these levels reflect the Bandwidth Efficiency and Signal Stability of the neural network. The need for support is not a measure of “pathological severity,” but rather the result of physical limitations in data processing:

- P-FIT bus resistance: physically, long-distance connectivity acts as a conductor. Diffusion Tensor Imaging (DTI) studies suggest that white matter integrity in ASD directly impacts processing speed (Wolff et al., 2012). Levels 2 and 3 may represent systems where the impedance of the P-FIT network (Jung and Haier, 2007) is too high for the signal load, causing energy dissipation.
- Thermal noise and saturation (E/I balance): support levels may also arise from an imbalance between excitation and inhibition (Rubenstein and Merzenich, 2003), a Signal-to-Noise Ratio (SNR) problem. If the glutamatergic “noise floor” rises without proper GABAergic inhibition, the system enters magnetic saturation, preventing the processing of new data.
- Sampling Density (Nyquist-Shannon): high-frequency sampling generates a data volume that exceeds the system’s Vaso-capacitance. More intense support levels may indicate a system operating at a sensory sampling frequency that challenges the Nyquist-Shannon Sampling Theorem (Shannon, 1948). According to the Intense World Theory (Markram and Markram, 2010), the brain generates more local information than it can integrate globally, resulting in unsustainable metabolic overhead. The cost of organizing this information (*W_erasure_*) would generate constant metabolic insolvency, regardless of external factors.

### 4.4 The ADHD dynamic: irradiation and exploratory entropy

In isolated ADHD, the architecture operates under a regime of irradiation. Due to an inconsistency in lateral inhibition (oscillating open gating) in the prefrontal cortex (Arnsten et al., 2012), the sensory signal is not confined but disperses through multiple circuits. This leads to unstable switching between the Default Mode Network and task-oriented networks (Castellanos and Proal, 2012), translating into multiple simultaneous processing threads. The system seeks to reduce uncertainty by constantly increasing environmental sampling (exploratory entropy).

Energy is dissipated across amplitude. The system opens multiple simultaneous processing threads to attempt to map environmental chaos. The problem is not a lack of focus, but an excess of targets. The metabolic cost (ATP expenditure) stems from constant context switching and the inability to maintain a stable resting state (Default Mode Network, DMN), requiring a continuous drain of ATP to sustain a vast processing network with low focal persistence.

### 4.5 The PTSD dynamic: hypervigilance and signal co-optation

In isolated PTSD, the processing system is co-opted by risk prediction. Neurobiological monitoring focuses exclusively on signals that may indicate a breach of homeostatic security.

The system operates in defensive overclocking. From a biophysical perspective, this represents a Signal Co-optation: the amygdala and the salience network hijack the available processing bandwidth to run continuous threat-detection subroutines (Rauch et al., 2006). Energy is drained to sustain a threat-prediction loop that frequently remains unresolved, keeping the structural substrate in a state of constant thermal readiness, even in the absence of real stimuli. The cost is driven by defensive hypervigilance, creating a state of Allostatic Load (McEwen, 2000) that rapidly depletes ATP reserves, narrowing the margin of solvency.

### 4.6 Flow dynamics (ADHD, ASD, and PTSD)

While the Open Gating serves as the input baseline, the divergence lies in the processing strategy. Whereas ASD consumes resources in an attempt to filter signal precision (systematization), ADHD consumes ATP in an attempt to map the noise amplitude (exploratory entropy), and PTSD consumes it in an attempt at threat prediction (defensive hypervigilance). The 2e Sentinel, however, operates in a synergy where solvency meta-prediction is not merely a response to noise, but a persistent attempt to sustain high-fidelity processing (see Table 5).

**Table 5:** Comparative matrix of predictive dynamics (HEPOE).

Feature	Sentinel (Giftedness)	ASD	ADHD	PTSD
State of being	Anticipation	Systematization	Exploration	Hypervigilance
Nature of difference	Fidelity	Connectivity (local integration)	Modulation (state instability)	Co-optation (security-focused substrate)
Gating dynamics	Open (fidelity demand)	Open (sensitivity)	Open (oscillatory instability)	Open (alert/vigilance)
Inference focus	Meta-predictive (solvency/pattern search)	Systematizing (order/predictability search)	Exploratory (novelty search)	Threat-driven (threat/danger search)
EWS output	“Knowledge of Collapse” (conscious anticipation)	Meltdown/shutdown (reaction to cease excessive input)	Impulsivity/escape (search for stimulus or scene change)	Flashback/dissociation (trauma avoidance reaction)
Solvency state	Solvent (architecture supports entropy)	Insolvent (processing exceeds structural capacity)	Erratic (high metabolic dispersion)	Deficitary (energy drained by hypervigilance)

Source: Brezolin and Freitas (2026).

## 5 Twice-exceptionality (2e): wave interference and architectural conflict

### 5.1 2e: wave interference and meta-prediction

**Figure 4:**
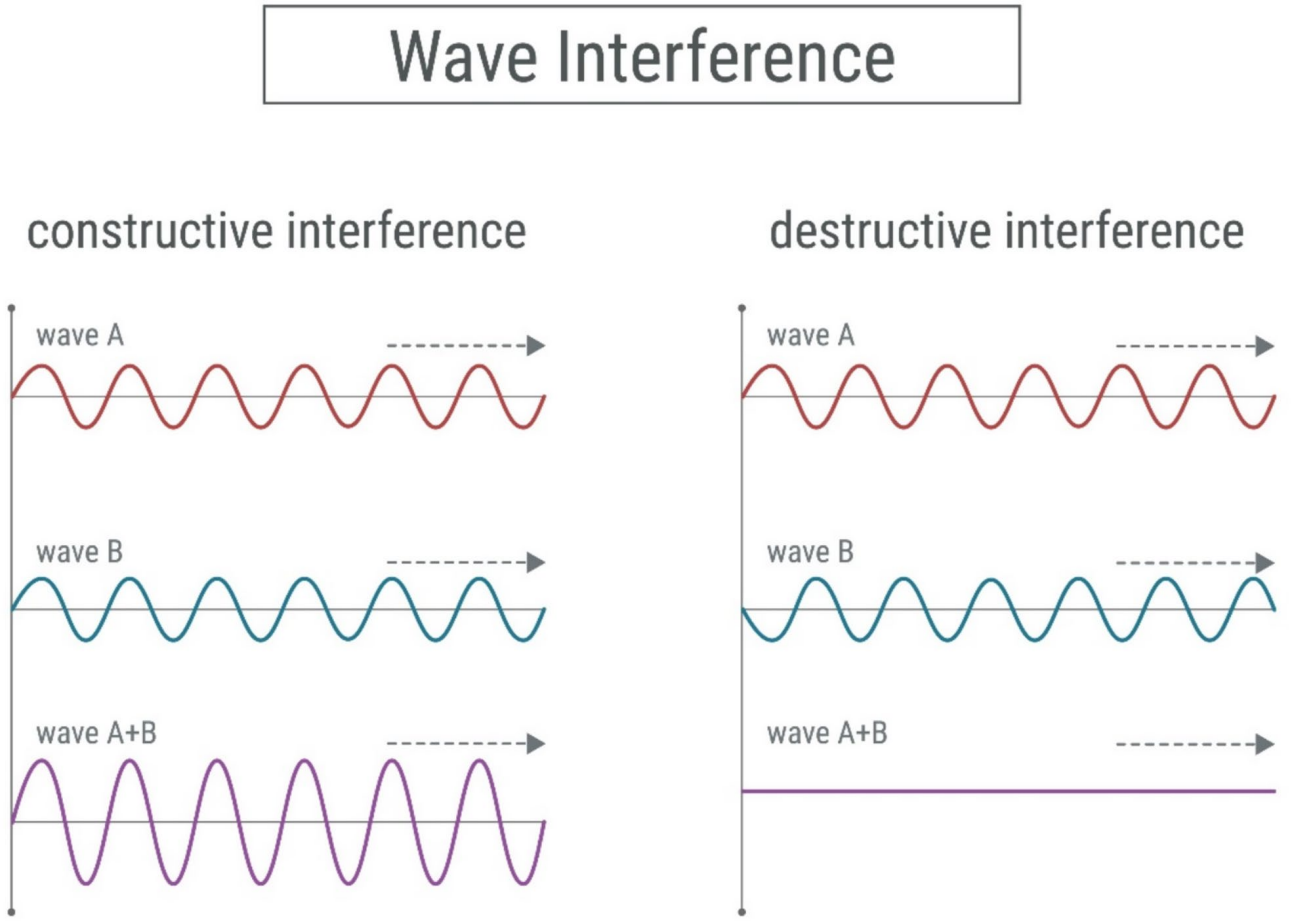
Principles of wave interference: constructive and destructive. The diagram illustrates the two fundamental types of wave superposition. *Left Panel: Constructive Interference (Amplification)*. Shows two sinusoidal waves, Wave A and Wave B, that are perfectly in phase (i.e., A’s peaks align with B’s peaks, and troughs with troughs). The result, Wave A + B, is a wave with the same frequency but amplified amplitude. Their energies combine, resulting in a more intense oscillation. *Right Panel: Destructive Interference (Phase Shift)*. Shows the same waves, Wave A and Wave B, but now in phase opposition (a phase shift of 180° or π radians). Wave A’s peak (crest) perfectly aligns with Wave B’s trough. The result, Wave A + B, is the total cancellation of the signal (a flat line). Their energies mutually cancel, resulting in silence or absence of oscillation. Source: Brezolin and Freitas (2026).

Only after understanding the Sentinel System as a high-fidelity neural architecture and the flow dynamics (ASD, ADHD, PTSD) as signal processing patterns (functional connectivity), can we define Twice-Exceptionality (2e). It is not the sum of diagnoses, but the phenomenon that occurs when the High-Fidelity structural substrate converges with these dynamics simultaneously (see Figure 4).

In this state, processing ceases to be linear and assumes the behavior of wave physics (Wave Interference):

- Constructive interference (amplification): occurs when irradiation peaks (ADHD) or sensory resonances (ASD) coincide in phase. For the Sentinel, which operates without data compression, this results in a “giant wave” of processing. The volume of raw information instantaneously exceeds the cooling and solvency capacity, leading to collapse due to systemic overload. The interference is not additive; it is exponential.
- Destructive interference (phase shift): this is the state of technical masking. The exploratory impulse (ADHD) or the need for systematization (ASD) operates in phase opposition. Externally, the forces cancel each other out, resulting in seemingly balanced behavior. However, thermodynamically, this signal cancellation does not eliminate the information cost; it transforms it into heat and entropy, according to Landauer’s Principle (Landauer, 1961), critically narrowing the bioenergetic solvency margin (Laughlin, 2001). In reality, this scenario requires an extreme computational load to harmonize divergent signals (Tononi, 2012) and resolve the precision conflict between models (Parr and Friston, 2017), imposing a state where the system consumes double the ATP to simultaneously process antagonistic vectors. This complexity in functional connectivity (Young et al., 2011) results in the “Engine Stall Cost”: the engine is at maximum rotation (overclocking), but the vehicle does not move (oxygen and ATP consumption rise drastically to maintain the stability of a system in conflict).

Neurobiologically, we propose that this wave interference corresponds to the desynchronization of neural oscillations. The high-fidelity neural architecture of the Sentinel tends to operate at Gamma frequencies (>30 Hz) to enable “Global Central Coherence.”

However, divergent dynamics (such as the stochastic noise of ADHD) introduce out-of-phase slow oscillations (Theta/Delta). The energy cost, therefore, arises from the disruption of Phase-Amplitude Coupling (PAC), forcing the system to consume excessive ATP in a futile attempt to resynchronize networks operating at incompatible temporal frequencies.

### 5.2 Sentinel + ASD

#### 5.2.1 Constructive interference (amplification): the granularity conflict

The Sentinel operates by seeking Entropy Reduction (extraction of global patterns/isomorphy). In contrast, the ASD mechanism operates by seeking Local Stability (reduction of variables and a search for immutability/detail).

When these two vectors collide, the Sentinel attempts to apply its “Entropy Reduction” (Macro) to the “Local Stability” (Micro) of ASD. Functions do not cancel out; rather, a scale superposition occurs (Young et al., 2011). The Sentinel Phenotype, designed to identify Global Patterns (Macro), applies its massive processing capacity to the Local Details (Micro) imposed by ASD neurobiology (Frith, 1989; Mottron et al., 2006).

The system does not view the detail merely as an isolated data point; it attempts to find a “Universal Link” within every sensory fragment. This is Recursive Amplification (Mandelbrot, 1982): the micro-detail is processed with the complexity and energetic weight of a macro-structure.

From this perspective, what is frequently labeled as Cognitive Rigidity manifests in the 2e individual as a functional adherence to the signal. It is not a volitional opposition, but a resolution imperative: the high-fidelity system cannot “de-focus” from the detail as long as the logical inconsistency remains unprocessed, prioritizing data integrity over the fluidity of the broader social context.

This establishes a clear boundary for differential diagnosis based on system teleology: clinical differentiation lies in the ‘Resolution Drive’. The Sentinel’s state persistence dissolves immediately upon verifying the isomorphic solution (the ‘Aha!’ moment), whereas in the ASD phenotype, state persistence serves the thermodynamic function of maintaining environmental stability, independent of logical resolution.

Consequently, a Data Load Exponentiation occurs. By treating every “pixel” of reality with the analytical depth reserved for the entire “frame,” the computational demand becomes fractal. The Meltdown is the physical response to this attempt to process the infinite within finite time, acting as a “Buffer Overflow”: working memory overflows not just from the volume of uncompressed sensory bytes, but from the impossibility of resolving the conflict between “Flow” (Sentinel) and “Fix” (ASD).

#### 5.2.2 Destructive interference (phase shift): masking through vector neutralization

Destructive interference in the Sentinel-ASD pair manifests as a state of apparent inertia that masks extreme metabolic activity. While the Sentinel’s structural substrate projects a goal of global coherence and the extraction of macro-structural patterns, the standard ASD connectivity dynamic imposes persistent anchoring in local details and signal stability through fixation rigidity.

When these vectors operate in phase opposition, a neutralization of functional output occurs: the Sentinel’s systemic expansion impulse and the ASD fixation anchor create a point of stasis. Thermodynamically, this phase-shift state results in a behavior of apparent balance or passivity, often mistaken for competence or emotional stability.

However, the system is actually processing a precision conflict between scales (Parr and Friston, 2017), consuming massive energetic resources in an attempt to decouple focus from detail without compromising the lossless integrity of the raw data. This is the deepest level of technical masking, where behavioral silence is the byproduct of internal neural friction that invisibly exhausts bioenergetic solvency for the external observer.

### 5.3 Sentinel + ADHD

#### 5.3.1 Constructive interference (amplification): high-fidelity multitasking

The Sentinel is a Long-Term Prediction machine. ADHD, due to dysregulation in dopaminergic gain (Volkow et al., 2009), is an Immediate Stimulus-Seeking machine (compensating for low cortical tone).

When these two vectors converge, an Exponential Arborescence synergy occurs. ADHD neurobiology acts as a stochastic generator of lateral associations (Branching). The Sentinel, consistent with its open sensory gating architecture, does not block these diversions; on the contrary, it integrates the ADHD “novelty” requirement and processes it with the Sentinel’s “prediction” depth.

The Sentinel Phenotype, whose primary function is Scenario Prediction, is forced to treat each of these branches as a potential future that needs to be calculated. The Lossless imperative compels the system to treat every intrusive thought or random association as a valid hypothesis deserving a full simulation (Non-Optimized Hyper-Threading). The individual is not “distracted”; they are attempting to execute multiple threads of complex reasoning in parallel.

The thermodynamic result is Thermal Throttling. The processor overheats not just from static load, but from Context Switching Friction: the energetic cost stems not from keeping the windows open, but from the internal heat generated by maintaining the resolution of each branch and restarting the reasoning process hundreds of times (Monsell, 2003). Each interruption forces a total reconstruction of the mental model (Landauer’s Cost of Erasure/Rewrite; Landauer, 1961), leading to rapid exhaustion. The Shutdown is the safety lock initiated to cease this destructive oscillation.

#### 5.3.2 Destructive interference (phase shift): analytical paralysis and the synchrony cost

In the encounter between the Sentinel architecture and ADHD dynamics, destructive interference takes the form of an operative paralysis resulting from the conflict between prediction and exploration. ADHD acts as a generator of irradiation across multiple associative threads, while the Sentinel demands that each of these branches be processed with absolute depth and long-term predictive validation. When the ADHD impulse to “initiate” is confronted by the Sentinel’s imperative to “conclude with perfection,” the signals enter phase opposition, resulting in a cancellation of executive movement.

Therefore, a critical distinction must be made regarding the system’s operational constraints. While the standard ADHD phenotype is characterized by a variance in inhibitory regulation, the Sentinel+ADHD interaction creates a “Secondary Executive Jam”: the executive architecture remains structurally intact but becomes functionally saturated by the massive volume of high-fidelity sensory inputs (buffer overflow). The resulting inertia is not a system failure, but a latency induced by processing overload.

This scenario is the classic example of the “Engine Stall Cost”: externally, the individual may appear unproductive or procrastinating, but internally, the structural substrate performs exhaustive work of constant rewriting (*W_erasure_*) and Context Switching under the Landauer limit (Landauer, 1961).

Energy is not translated into action but dissipated in a frustrated attempt to organize exploratory chaos into zero-error models. One vector cancels the other: the search for novelty is locked by the rigor of analysis. The system consumes oxygen and ATP at overclocking levels just to maintain the precarious balance between the desire for expansion and the rigor of simulation.

The somatic consequence of this state of “high static rotation” is the accumulation of metabolic byproducts, specifically Lactate. The resulting local lactic acidosis, combined with systemic inflammation from oxidative stress, provides the biophysical basis for the diffuse pain and chronic fatigue (similar to fibromyalgia) frequently reported by 2e patients, validating the complaint of physical pain without an apparent lesional cause.

### 5.4 Sentinel + PTSD

#### 5.4.1 Constructive interference (amplification): processing diversion (signal bias)

The Sentinel Phenotype natively operates in the pursuit of Entropy Reduction (understanding the unknown). PTSD imposes a diametrically opposite objective: Immediate Survival (fearing the unknown). The system ceases to seek “Isomorphies” (Patterns) and begins seeking exclusively “Anomalies” (Threats).

When these vectors converge, they represent an Algorithm Co-optation. The Gating (filtering), which in the Sentinel is naturally open for curiosity, becomes a “Reactive Gating”: maximum openness is maintained but conditioned to fear signatures (Litz et al., 2009). The system utilizes the Sentinel’s massive processing capacity not for innovation, but to run Worst-Case Scenario Simulations in a continuous loop. “High-Fidelity” becomes a curse: the individual perceives danger micro-signals that no one else sees, logically validating their hypervigilance. The Sentinel’s high-fidelity neural architecture, capable of detecting subtle patterns, is reprogrammed to operate exclusively in security anomaly detection.

The thermodynamic result is Chronic Allostatic Overload (McEwen, 1998). Energy that should fuel the prefrontal cortex (executive function/creativity) is permanently diverted to the limbic system and the HPA Axis. The collapse here is neither a Meltdown (purge) nor a Shutdown (inertia), but Grid Tension Exhaustion: the biological battery becomes “conditioned” (degraded) by perpetually operating at emergency voltage.

Beyond ATP depletion, the Allostatic Load in this scenario is exacerbated by the toxicity of the HPA Axis. The system, operating in constant threat prediction, maintains elevated basal levels of Cortisol to mobilize emergency glucose. Chronic exposure to glucocorticoids results in neurotoxicity, compromising the integrity of the hippocampus and working memory, creating a feedback loop where memory failure increases the sense of insecurity and predictive demand.

#### 5.4.2 Destructive interference (phase shift): stasis through gating antagonism

Destructive interference in the Sentinel-PTSD context represents the apex of thermodynamic inefficiency due to signal diversion. It is fundamental to note that both states operate under an open gating regime, yet with antagonistic operational objectives. While the Sentinel’s high-fidelity neural architecture utilizes sensory openness for isomorphy extraction and exploratory curiosity, the functional connectivity dynamics of PTSD use the same openness to sustain reactive hypervigilance.

The interference occurs when the impulse for signal comprehension (Sentinel) collides with the imperative of stimulus avoidance (PTSD). The structural substrate continues to capture information in High-Fidelity (Lossless), but the defensive processing pattern attempts to neutralize this signal before it reaches executive consciousness to preserve homeostatic security. Energy is not used to process reality, but to maintain the barrier between high-fidelity perception and the traumatic emotional load.

The result is a Destructive Flow Interference: the wave crests of curiosity are canceled out by the phase troughs of avoidance. The system maintains the high cost of thermal readiness and the ATP consumption of PTSD, but the information is not integrated as knowledge. This state of “High-Voltage Standby” consumes bioenergetic solvency to sustain reactive silence, protecting the structural substrate from emotional collapse but preventing fluid functionality. The individual becomes a high-performance processor that detects everything but is prevented from acting upon what it perceives.

### 5.5 Intensified convergence and systemic insolvency

Intensified Convergence describes the state of saturation in which the Sentinel’s high-fidelity neural architecture is simultaneously subjected to multiple conflicting functional connectivity vectors: recursive amplification (ASD), stochastic irradiation (ADHD), and reactive monitoring (PTSD).

In this scenario, the system’s Bandwidth is entirely consumed by the effort of processing interferences across multiple scales, reducing the capacity for external cognitive operations to zero. The system ceases to process external information and shifts its resource allocation exclusively to maintaining structural integrity.

#### 5.5.1 The virtual machine: the computational cost of masking

To avoid immediate functional collapse, the system activates a Normality Emulation Protocol, commonly known as Masking (Hull et al., 2017; Attwood, 2007). From an engineering perspective, masking operates as a high-consumption “Virtual Machine” executed over the native connectivity pattern. The system is forced to intellectually calculate, via the Prefrontal Cortex, responses that should be processed through intuitive heuristics. This process converts social interaction into a Unilateral Energy Drain, where the cost of maintaining the emulation prevents the solvency of other vital functions.

#### 5.5.2 Burnout as chronic structural failure

The thermodynamic result of this convergence is not an acute event, but a chronic structural failure, identified in the literature as Autistic Burnout (Raymaker et al., 2020). In the 2e context, we propose that this state be reclassified as Systemic Metabolic Insolvency.

In this phase, a critical depletion of astrocytic glycogen and dopamine reserves occurs, resulting in a “loss of function” for protection. Access to higher-order abilities and the P-FIT network (Jung and Haier, 2007) is temporarily blocked (intelligence “shuts down”) because the meta-prediction system identifies that continuing high-fidelity processing would lead to irreversible thermal and excitotoxic damage. The structural substrate enters Core Preservation Mode, where the little remaining energy is diverted exclusively to basic autonomic functions.

#### 5.5.3 The “imposter phenomenon” as predictive overload: an entropic byproduct in Sentinel-specific architectures

Traditional psychology and clinical psychiatry frequently categorize the “Imposter Syndrome” in Sentinel Phenotypes and twice-exceptional (2e) populations as a psychological deficit, a manifestation of low self-esteem, chronic insecurity, or emotional dysregulation. While these psychological factors may affect the general population, the HEPOE framework identifies a distinct neurobiological variant specific to the Sentinel Phenotype. Under the HEPOE framework, the Sentinel’s experience could be reframed as an additional, non-psychological layer of predictive friction: it is not an emotional flaw, but rather a direct thermodynamic byproduct of predictive overload within high-fidelity neural architectures.

Beyond common emotional insecurity, the Sentinel Phenotype continuously computes environmental entropy (*H(E)*) with extreme granular precision. Operating at a high spatial–temporal resolution, the system inherently maps an exponential number of edge cases, hidden variables, and potential systemic failures before executing an action or consolidating a predictive model. Because the architecture runs massive parallel simulations of “what could go wrong” to ensure ultimate homeostatic survival, it generates a continuous stream of predictive error alerts (Δ*P*).

Clinically, this high-volume error signaling is often internalized and misinterpreted by the individual as standard “insecurity” or “self-doubt”. Thermodynamically, however, it functions as an advanced Homeostatic Risk-Prevention Mechanism. The system is merely stating: the entropic complexity is too high; do not close the predictive model yet until all variables are mitigated and the risk of breaching the solvency threshold is neutralized.

Furthermore, this phenomenon is exacerbated by the paradox of high cognitive resolution. As the predictive horizon expands, the awareness of the “unknown” scales exponentially. The Sentinel evaluates their own performance against a highly optimized, theoretically perfect model that their neural architecture can internally visualize, rather than against standard baseline metrics. When the individual attempts to recalibrate these high-fidelity insights to function within standard operational timelines, they must actively suppress or delete a vast amount of computed risk data. The act of ignoring these advanced predictive models triggers a severe Landauer’s penalty (Landauer, 1961) (*W_erasure_* in HEPOE). The sheer thermodynamic weight of this forced cognitive pruning generates profound entropic friction, which the individual experiences as a persistent feeling of intellectual inadequacy, fraudulence, and exhaustion, known as the classic, yet physically misunderstood, Imposter Phenomenon within this specific population.

#### 5.5.4 Other highly prevalent behavioral traits in the Sentinel and 2e population

Beyond the Imposter Phenomenon, this predictive and thermodynamic framework provides a unified physical mechanism for other highly prevalent, yet poorly understood, behavioral traits in Sentinel Phenotypes and 2e populations.

For instance, Paralyzing Perfectionism can be mathematically modeled as a resistance to data compression: an active biological avoidance of the Landauer penalty (Landauer, 1961) (*W_erasure_* in HEPOE) required to downgrade a high-fidelity internal model into a lower-resolution external output. To the external observer, it appears as behavioral rigidity; to the physics of the brain, it is an energy conservation strategy designed to prevent the massive ATP drain of rewriting intact models.

Similarly, the acute Justice Sensitivity frequently observed in these individuals is not merely a moral stance, but a severe intolerance to structural inefficiencies. The high-resolution architecture detects illogical, hypocritical, or inefficient social frameworks as massive Narrative Dissonance (Δ*P*), forcing the neural system to expend extreme computational energy attempting to resolve an inherently broken external logic.

## 6 The phenomenology of collapse: systems thermodynamics

Historically, terms such as Meltdown and Shutdown have been exclusive to the lexicon of autism to describe emotional dysregulation. In the HEPOE approach, we reappropriate these terms to describe Systems Physics events: they are not functional defects, but rather protection and preservation protocols for the neural architecture, resulting from the clash between distinct operational objectives (e.g., expansion vs. anchoring).

### 6.1 The lactate-glutamate crisis (supply bottleneck)

The core vulnerability of the system is logistical in nature. Informational hyper-processing depletes astrocytic glycogen (Brown and Ransom, 2007) at a rate exceeding vascular replacement capacity, representing a classic “Just-in-Time” delivery failure. Simultaneously, the drop in ATP levels prevents efficient glutamate reuptake by astrocytes, shifting the burden from functional processing to structural survival.

In high-fidelity architectures, this metabolic demand is not only a neuronal burden but a glial one. We propose that Systemic Metabolic Insolvency is primarily a failure of astrocytic support. When the rate of glutamate accumulation outpaces the recycling capacity of the glia, the resulting oxidative stress creates a ‘recovery lag’, explaining why 2e burnout requires prolonged periods of low entropy for structural restoration.

The accumulation of this neurotransmitter in the synaptic cleft triggers an imminent risk of excitotoxicity, mediated by a massive calcium influx and subsequent oxidative stress, which threatens the physical integrity of neurons due to the extremely high metabolic cost of information transmission (Laughlin, 2001). This vascular saturation and the imbalance between lactate and glutamate establish the physical tolerance limit of the structural substrate, whose adaptive protective responses (safety protocols) will be detailed hereafter in the forms of Meltdown and Shutdown.

This collapse is not merely functional but is rooted in substrate science: the high-fidelity neural architecture operates under load conditions that challenge the tolerance limits of the biological material, where the exhaustion of astrocytic glycogen represents a breach of the structural integrity required for system maintenance.

#### 6.1.1 Encoding imperative vs. sensory saturation

It is critical to distinguish between pure sensory saturation and the “Encoding Imperative.” In High-Fidelity architectures, distress arises not merely from the intensity of the stimulus, but from a systemic compulsion to extract mathematical patterns or predictive coherence from stochastic noise. While typical overload is a volume-threshold issue, the Sentinel’s burden is a computational deadlock: the brain is intrinsically driven to process entropy, consuming massive bioenergetic resources in a search for isomorphy within non-convergent signals. Consequently, tools that reduce environmental entropy, such as Active Noise Cancelling (ANC) devices, serve as essential hardware-level mitigators, directly lowering *H(E)* and preserving the system’s metabolic solvency for executive functions. This leads to a sustained state of high internal entropy without resolution.

### 6.2 Meltdown: buffer overflow protocol

Under the HEPOE Theory, the Meltdown must be reclassified not as a behavior, but as an Entropy Purge event. When working memory reaches total saturation (Buffer Overflow) due to an excess of uncompressed data (*H(E)*), the system activates intense motor mechanisms (screaming, rapid stereotypies) to dissipate accumulated kinetic energy.

This reaction is, therefore, a biological attempt to reduce the internal load, functioning as an imperative thermodynamic “relief valve” designed to prevent irreversible neuronal damage from excitotoxicity (Laughlin, 2001).

### 6.3 Shutdown: “safe mode” protocol

Diametrically opposed, the Shutdown is the biological equivalent of Thermal Throttling in processors. Upon detecting critical energy levels, the brainstem executes a severe Underclocking: it cuts the power supply to high-consumption areas (Broca’s Area/Language and Motor Cortex) to preserve the vital core. The resulting inertia is the only way to maintain the system’s temperature within safe operational limits.

## 7 Analysis: the cost of fidelity

### 7.1 Predictive moral injury (original concept)

Unlike classical moral damage in the legal sphere or reactive ethical suffering, Predictive Moral Injury (Brezolin and Freitas, 2026) is defined by HEPOE Theory as the anticipated and systemic suffering arising from the Sentinel’s high-fidelity processing.

This phenomenon occurs when the hardware, upon detecting isomorphies and patterns of collapse in social, ethical, or organizational systems, projects the deleterious outcome long before its factual manifestation. The injury resides in the impossibility of “unseeing” the pattern and in the allostatic load generated by the attempt to signal a danger that the environment (characterized by low entropy or simplified processing) is not yet capable of perceiving.

Therefore, Predictive Moral Injury is the burden of predictive precision in a noisy medium.

### 7.2 The ontology of Predictive Moral Injury

The proposition of the term Predictive Moral Injury as a pillar of HEPOE Theory is grounded in the need to name the systemic illness phenomenon that precedes the factual event. Unlike reactive moral damage, resulting from an already consummated aggression, predictive injury is the lesion generated by the continuous sustainment of a dissonant truth. The metabolic cost of sustaining a high-fidelity internal model while the environment operates on a degraded model (low resolution) results in a bioenergetic synchronization failure.

The foundation of Predictive Moral Injury dialogues with Dejours (2007) concept of Ethical Suffering, which describes the impact of betraying individual values within the labor environment. However, HEPOE Theory expands this notion by integrating it with the Moral Injury described by Shay (2014) and the clinical frameworks of moral repair (Litz et al., 2009).

While in classical literature injury is often seen as a response to a past event, in the Sentinel, it assumes an anticipatory character. As Bauman and Donskis (2014) state regarding the “moral blindness” of institutions, the Sentinel suffers biological injury at the exact moment they detect the isomorphy of collapse that the system refuses to see, transforming ethical prediction into an unbearable allostatic load.

#### 7.2.1 The cause: axiological conflict and isomorphy

The Sentinel, through high-fidelity sampling, prematurely detects isomorphies of corruption, injustice, or systemic failure in organizational and social environments. Predictive Moral Injury is born the moment the hardware identifies the error pattern, but the environment (low resolution) denies, ignores, or punishes the signaling. This injury is exacerbated in environments that lack psychological safety (Edmondson, 1999), preventing the resolution of the detected error. Morality here is not a subjective code of conduct, but conformity with logical and systemic truth.

#### 7.2.2 The mechanism: somatization of denied uncertainty

When the environment prevents the Sentinel from acting on a prediction (Action Constraint), the Free Energy cannot be minimized. The brain, unable to update the external world to match its model, attempts to update its internal model but fails due to the high fidelity of its perception. This triggers a recursive, high-frequency reverberation loop we term the “Spinning in Place” Cycle: neural circuitry re-runs the prediction error correction algorithm repeatedly without resolution.

##### 7.2.2.1 The thermodynamic cost (the physics)

This phenomenon is grounded in the Second Law of Thermodynamics and quantified by Landauer’s Principle (Landauer, 1961), which dictates that information erasure is a dissipative process.

For the Sentinel brain (HEPOE), the detection of isomorphism is the default state. When a Sentinel identifies a systemic lie but is forced to ignore it, the brain is biologically compelled to “erase” a valid bit of high-fidelity information. The minimum heat (*Q*) dissipated is:


$$Q\geq k_{B}\;T\;\ln\;2$$

Where:

- *Q*: The minimum amount of heat dissipated into the environment per erased bit of information [Unit: Joules (J)].
- *k_B_*: The Boltzmann constant (1.38 × 10^−23^ J/K) (Boltzmann, 2015) [Unit: Joules/Kelvin (J/K)].
- *T*: The absolute temperature of the biological system [Unit: Kelvin (K)].
- ln 2: The natural logarithm of 2 (representing the binary state erasure) [Unit: Dimensionless].

Therefore, “masking” one’s intelligence is not merely a social act; it is a physical process of information destruction that dissipates heat.

##### 7.2.2.2 The biological cascade (the damage)

To sustain this high-frequency “erasure loop” and manage the thermal excess, the system enters a pathological state characterized by the accumulation of neurotoxic byproducts:

- Alert Hypermetabolism: ATP consumption is directed toward obsessive simulations of conflict resolutions that the environment does not allow to be resolved. This state triggers a chronic demand on the system’s regulatory mechanisms, leading to the wear and tear known as allostatic load (Sterling and Eyer, 1988). Biologically, this rapid synaptic firing drives the system into anaerobic glycolysis, leading to the accumulation of Lactate and local acidosis. In this state, Moral Blindness emerges not as a psychological defense, but as a thermodynamic survival strategy. The hardware is biologically driven to “shut down” ethical-moral perception to prevent total system collapse caused by thermal excess and critical ATP depletion (Laughlin, 2001). This biological imperative to minimize variational free energy (Friston, 2010) prioritizes core preservation over the metabolic cost of high-level signal processing. This phenomenon aligns with the concept of ‘Adiaphorization’ in liquid modernity (Bauman and Donskis, 2014). In the HEPOE framework, what Bauman and Donskis (2014) describe as moral insensitivity is revealed as an ‘anaesthetized’ state, representing a systemic response to environments where entropy exceeds the hardware’s resynthesis capacity;
- Allostatic Erosion: hardware expression depends directly on the individual’s Allostatic Load (Sterling and Eyer, 1988). An environment of high entropy and low ethical nutrition drains the Sentinel’s ATP just for biological survival maintenance, preventing creative output and generating what common sense interprets as “social failure.” Under HEPOE, this “failure” is not a hardware flaw, but the inevitable logistical result of operating in a crisis regime;
- Cellular Damage (Excitotoxicity): this may lead to quantifiable somatic damage driven by Excitotoxicity. The loop floods the synaptic cleft with Glutamate (the primary excitatory neurotransmitter), causing oxidative stress and inflammatory processes. This leads to neurovegetative disorders and the collapse of energy resynthesis.

##### 7.2.2.3 The protective response: adiaphoric throttling (“brain fog”)

At a critical threshold, to prevent permanent somatic damage, the Sentinel brain activates a Biological Downregulation Mechanism.

We term this “Type II Sentinel Fog” (Adiaphoric Throttling). Unlike standard fatigue (Type I), which is fuel depletion, this is a protective deceleration of synaptic velocity, which is analogous to thermal throttling in high-performance processors, to stop the production of heat and toxins. The Sentinel becomes “slow” not because they lack capacity, but because their hardware is actively braking to prevent a meltdown caused by environmental incoherence.

##### 7.2.2.4 The solvency of information flow

Finally, we formalize this limit through the Predictive Solvency Inequality. For the system to remain functional, the Predictive Fidelity Capacity (*C*) must satisfy:


$$C\geq H(E)+\mathrm{\Delta P}+W_{\text{erasure}}$$

Where:

- *C* (High-Fidelity Capacity): the maximum rate of error-free information processing supported by the neural channel [Unit: bits/min].
- *H(E)* (Environmental Entropy): the raw volume of unfiltered sensory bits [Unit: bits/min].
- Δ*P* (Predictive Precision): the computational bandwidth required for sustaining zero-error internal models [Unit: bits/min].
- *W_erasure_* (Erasure Work): the processing bandwidth actively hijacked or consumed by the thermodynamic penalty of suppressing valid predictions (Landauer’s Penalty) (Landauer, 1961) [Unit: bits/min].

**Figure 5:**
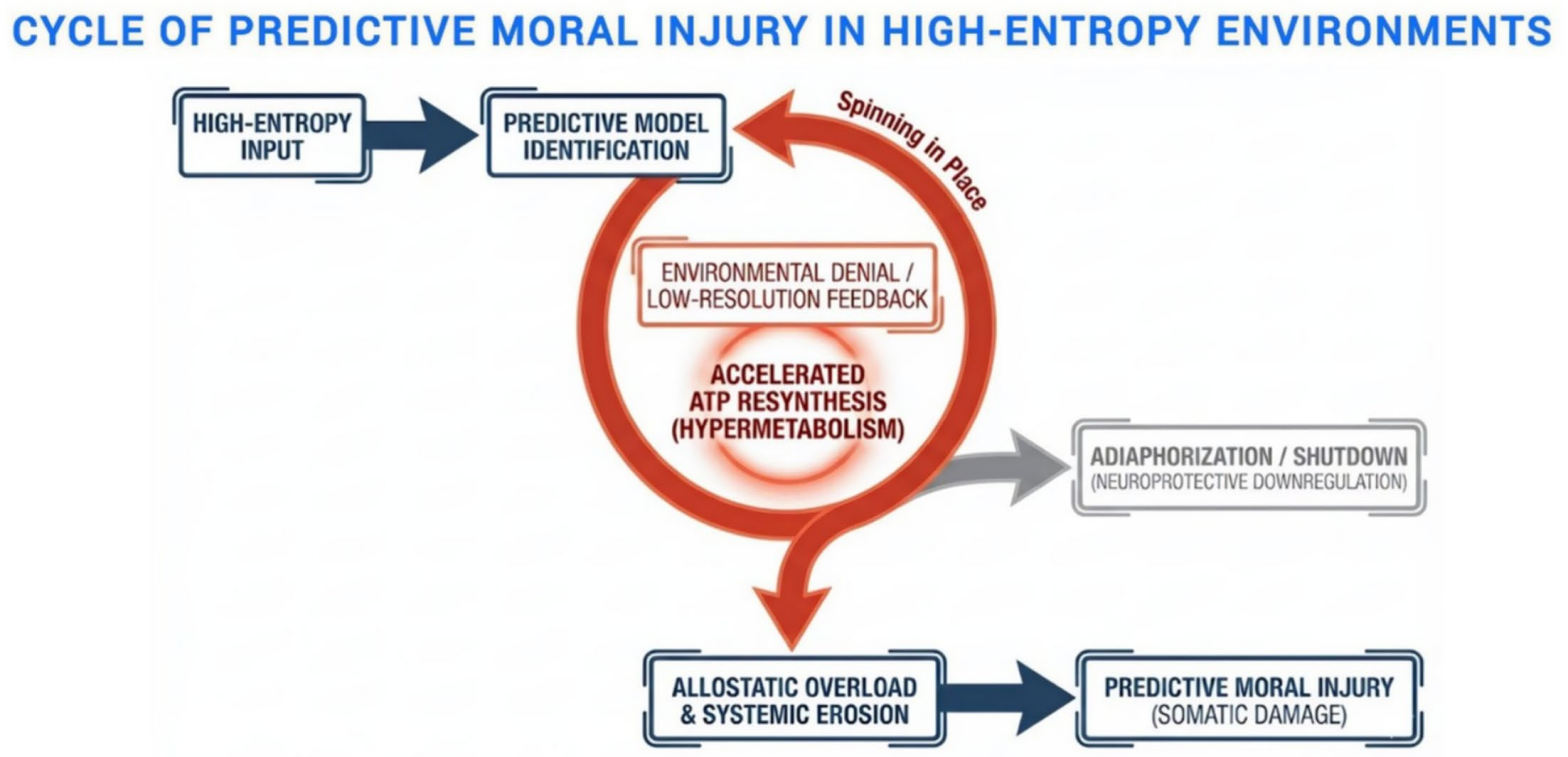
The “Spinning in Place” dynamics and Predictive Moral Injury. The schematic illustrates the bioenergetic collapse of the Sentinel hardware when operating in low-resolution environments. Input: the system detects a high-fidelity pattern of systemic failure, defined as Ethical or Systemic Collapse Isomorphy. Conflict: the environment denies or punishes the signal, preventing corrective active inference. Hypermetabolism: the hardware enters a recursive loop of predictive recalibration, the “Spinning in Place” cycle, consuming excessive ATP to resolve the dissonance. Outcome: this trajectory potentially involves somatic damage, defined as Predictive Moral Injury, due to Allostatic Overload, or a neuroprotective shutdown known as Adiaphorization (Bauman and Donskis, 2014). Source: Brezolin and Freitas (2026).

If the environmental entropy is too high, the equation becomes unbalanced (*E_met_ > ATP_max_*), forcing the system into the collapse described in the HEPOE Theory (see Figure 5).

##### 7.2.2.5 Note on dimensional alignment and isomorphic transfer functions

To ensure rigorous dimensional consistency within the Predictive Solvency Inequality (*C* ≥ *H(E)* + Δ*P* + *W_erasure_*), we define the informational work of erasure (*W_erasure_*) through a thermodynamic transfer function. While the system’s capacity (*C*) and environmental entropy (*H(E)*) are measured as informational flux (bits/min), the metabolic cost of suppression has a dynamic thermal nature.

The term *W_erasure_* is therefore derived as follows:


$$W_{\text{erasure}}=\frac{{\dot{Q}}_{\text{erasure}}}{\epsilon_{\mathrm{sys}}}$$

Where:

- *W_erasure_*: the processing bandwidth actively hijacked or consumed by the thermodynamic penalty of suppressing valid predictions (Landauer’s Penalty) (Landauer, 1961) [Unit: bits/min].
-
$\dot{Q}$ *_erasure_* (Entropy Dissipation Rate): represents the instantaneous flux of thermal energy generated by the active suppression of valid predictive models (Masking) [Unit: Joules/min (J/min)].
- ϵ*_sys_* (Universal State Strain Coefficient): represents the specific energy required to displace the neural substrate from homeostatic equilibrium to register or erase a single bit of information [Unit: Joules/bit (J/bit)].

**Figure 6:**
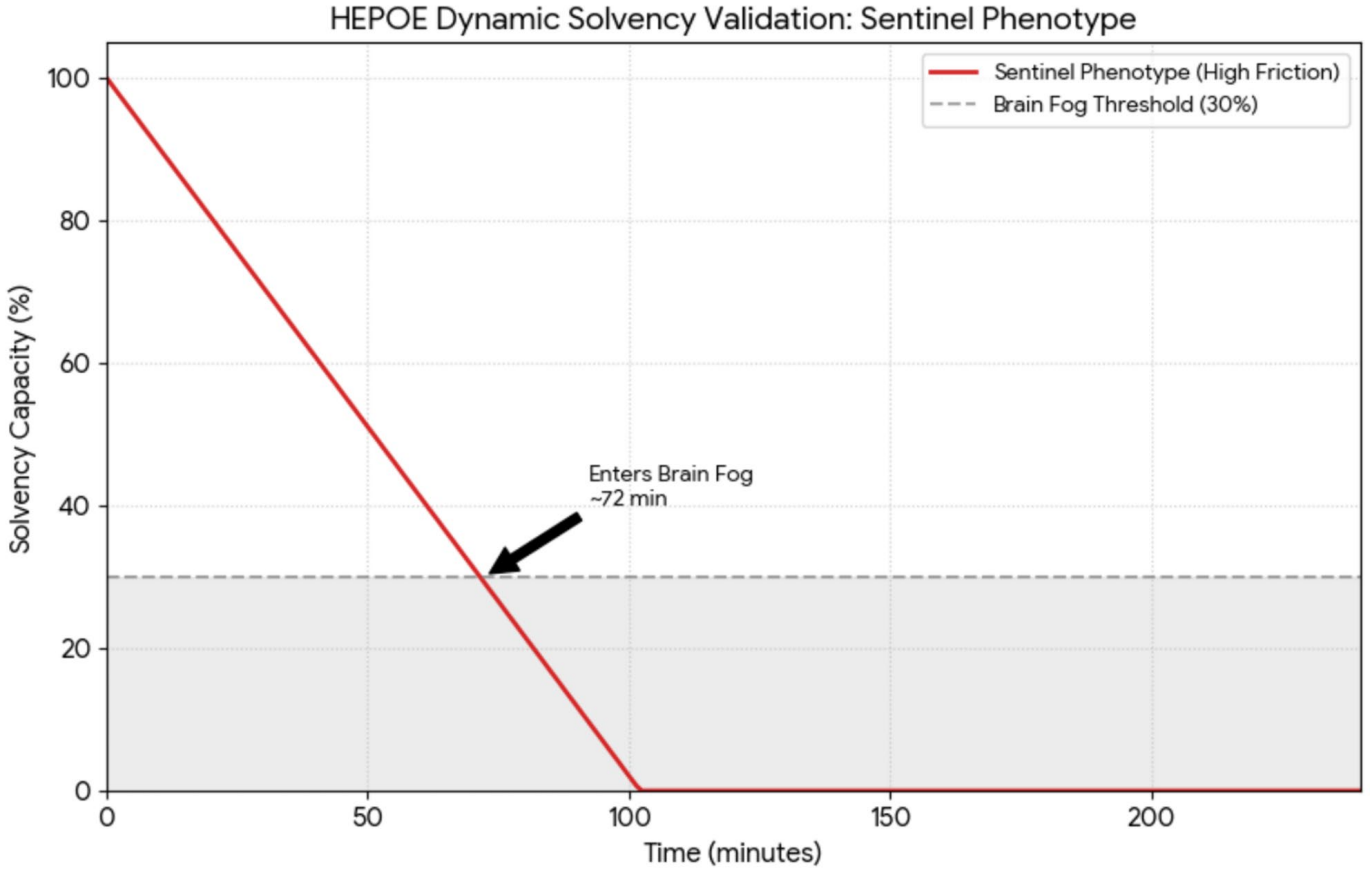
HEPOE dynamic solvency validation: Sentinel Phenotype. Solid Red Line (High Friction Sentinel). Illustrates the rapid depletion of solvency capacity over time for a Sentinel operating under high systemic friction (Ω > 1.0). Dashed Gray Line (Brain Fog Threshold). Demarcates the critical 30% solvency limit. Shaded Gray Area. Represents the insolvency zone where Adiaphoric Throttling (brain fog) is triggered to prevent structural excitotoxic damage. Annotation. Highlights the specific Breakpoint, demonstrating that the system enters brain fog at approximately 72 min of continuous high-fidelity processing. Source: Brezolin and Freitas (2026).

Physically, this formulation allows for the seamless integration of Landauer’s Principle (Landauer, 1961) into the HEPOE framework, demonstrating that “Brain Fog” (Type II) is a dynamic throttling of the bits/min budget. In this state, the Sentinel brain does not lose raw capacity (*C*). Rather, the system is forced to divert increasing portions of its bits/min budget solely to manage and dissipate the heat flux ( $\dot{Q}$ *_erasure_*) generated by the friction of maintaining high-fidelity predictive models in low-resolution environments (see Figure 6).

#### 7.2.3 Justification of nomenclature

The term “Moral” was maintained to denounce that the cause of the Sentinel’s physical injury is typically linked to an external violation of integrity. Labeling this phenomenon as “stress” or “anxiety” would be a technical imprecision that would blame the hardware for the environment’s toxicity. Predictive Moral Injury is, therefore, the physical record of a truth that the world is not yet ready to process.

#### 7.2.4 Distinction between empathic resonance and predictive injury

It is fundamental to differentiate Predictive Moral Injury from simple empathy or emotional contagion. While empathy is an affective response to another’s state, Predictive Injury is a technical response to pattern dissonance. The Sentinel can suffer moral injury even in impersonal situations (such as analyzing a defective engineering project or an unsustainable public policy). The injury occurs through exposure to ethical entropy: the biological cost of processing a structural failure that the environment insists on maintaining. Therefore, the lesion does not stem from “feeling,” but from “knowing” and the impossibility of acting upon the predictive signal of collapse.

### 7.3 Computational validation and bioenergetic formalism

To address the necessary operationalization of the Sentinel Phenotype and validate the thermodynamic costs described in Section 4.2, we transition from phenomenological descriptions to a rigorously quantified thermodynamic framework. We employed agent-based modeling (Python/NumPy) to create a “Digital Twin” comparing two neural phenotypes under identical environmental conditions.

#### 7.3.1 Dimensional analysis and the thermodynamic cost of erasure

A fundamental challenge in neurothermodynamics is bridging the scalar gap between microscopic informational erasure and macroscopic ATP hydrolysis. According to Landauer’s Principle (Landauer, 1961), the minimum energy dissipated to erase one bit of information is *Q* ≥ *k_B_ T* ln 2, where *k_B_* is the Boltzmann constant (Boltzmann, 2015) and *T* is the absolute temperature (310 K for the human body).

However, suppressing a high-fidelity cognitive insight (Masking) is not a single-bit operation but an emergent macroscopic event recruiting distributed cortical networks (P-FIT). To ensure dimensional consistency between information theory and bioenergetics, we introduce the Neural Amplification Coefficient (*N_amp_ ≈* 10^20^). This coefficient acts as the conversion function, accounting for the massive thermodynamic inefficiency of synaptic transmission and the fact that neurotransmitter reuptake management involves molar scales (Avogadro’s number).

Therefore, the total thermodynamic work of erasure (*W_erasure_*) required to sustain Active Inference Suppression is quantified as:*Q˙_erasure_* = (*Bits_erased_*⋅ *k_B_ T*ln 2). *N_amp_*

Where:

- *Q˙_erasure_*: the total macroscopic thermodynamic power (rate of work) of erasure required to sustain Active Inference Suppression (Landauer’s Penalty) (Landauer, 1961) [Unit: J/min].
- *Bits_erased_*: the rate of cognitively suppressed information [Unit: bits/min].
- *k_B_*: the Boltzmann constant (1.38 × 10^−23^ J/K) (Boltzmann, 2015) [Unit: Joules/Kelvin (J/K)].
- *T*: the absolute temperature of the biological system [Unit: Kelvin (K)].
- *N_amp_*: the Neural Amplification Coefficient, bridging the microscopic quantum event of bit erasure to the macroscopic molar scale of ATP hydrolysis [Unit: Dimensionless, ≈ 10^20^].

In this equation, *Bits_erased_* represents the volume of cognitively suppressed information, which is multiplied by the base thermodynamic cost per bit (*k_B_ T* ln 2) and scaled to the biological molar level by *Namp*, yielding the total macroscopic work required to sustain behavioral masking.

The order of magnitude for the Neural Amplification Coefficient (*N_amp_* ≈ 10^20^) is heuristically derived from the multiplication of the brain’s intrinsic thermodynamic inefficiency (operating ≈ 10^6^ above the Landauer limit; Laughlin, 2001) by the massive synaptic recruitment required for active macroscopic inhibition across the P-FIT network (≈ 10^9^ simultaneous synapses; Jung and Haier, 2007), scaled to the stoichiometric molar level of ATP hydrolysis.

**Figure 7:**
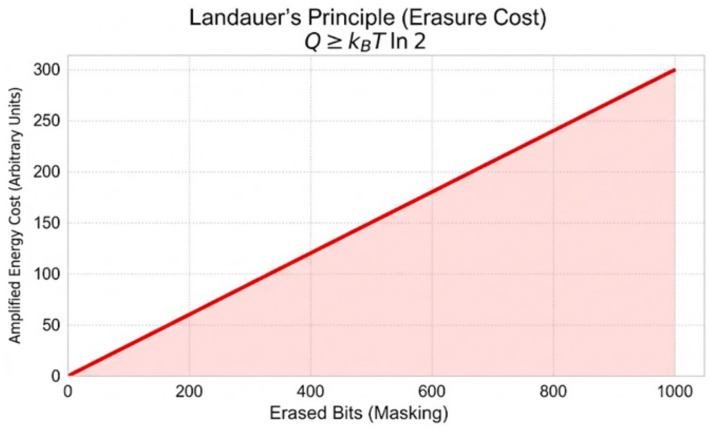
Landauer’s Principle (Erase Cost). Based on *Q ≥ k*_B_*T* ln 2. The graph illustrates the direct linear relationship between the quantity of suppressed insights (*W_erasure_*) and thermal energy dissipation. This validates the hypothesis that social camouflaging (Masking) imposes a physical metabolic penalty, contributing to systemic exhaustion. Source: Brezolin and Freitas (2026).

This represents the “Systemic Lie” test, validating the application of Landauer’s Principle (Landauer, 1961) to psychology. The simulation demonstrates that the act of Masking (pretending not to see a pattern) is not passive; it is active thermodynamic work. The more the Sentinel must suppress valid insights to adapt socially, the more physical heat their brain dissipates, leading to local hyperthermia. This dimensional translation indicates that social camouflaging and error suppression impose a severe, quantifiable physical penalty, directly contributing to systemic exhaustion (see Figure 7).

#### 7.3.2 Simulation parameters

The computational stress test was configured using the following parameters:

- Environment: a continuous 120-min (2-h) session of exposure to a high-uncertainty data stream (*H* ≈ 120 bits/min).
- Agent A (control/neurotypical): configured with efficient *Gating* (filters 70% of stimuli) and low Erasure cost (passive social compliance).
- Agent B (HEPOE Sentinel): configured with Open Gating (simulating the Leaky Thalamus dynamics [Schmitt and Halassa, 2017]; processes 95% of stimuli) and high *Masking* necessity (active suppression of detected isomorphisms).
- Physical variables: Boltzmann constant (*k_B_*) (Boltzmann, 2015) and Body Temperature (310 K) were applied to calculate the Landauer cost (Landauer, 1961).
- Thermodynamic constraints: the model applies the *W_erasure_* equation formulated in Section 4.3.1. While standard body temperature (*T* = 310 K) is used, any local hyperthermia caused by high metabolic demand would increase the base Landauer limit (Landauer, 1961) (*Q ≥ k_B_ T* ln 2). Therefore, our simulated dissipation represents a conservative estimate of the neurocognitive collapse.

#### 7.3.3 Glycogen buffering and the *T_acute_* constraint

To formalize the temporal limits of high-fidelity processing, we anchor the HEPOE Breakpoint to empirical neuroenergetic stoichiometry. Advanced Magnetic Resonance Spectroscopy indicates that the healthy human brain stores approximately 8.0 *μmol/g* of glycogen (Öz et al., 2015). Given a basal cerebral metabolic rate of glucose (*CMR_glc_*) of approximately 0.4 *μmol/g/min* (Laughlin, 2001), the Acute Stress Window (*T_acute_*) acting as the primary astrocytic energy buffer is mathematically constrained:


$$T_{\text{acute}}=\frac{\text{Glycogen Reserve}}{\text{Metabolic Rate}}=\frac{8.0\;\mu \mathrm{mol}/g}{0.4\;\mu \mathrm{mol}/g/\min}=20\;\text{minutes}\;(\text{Basal})$$

Where:

- *T_acute_*: the temporal limit of the primary astrocytic energy buffer [Unit: min].
- Glycogen reserve: the *in vivo* molar concentration of human brain glycogen [Unit: μ *mol*/g].
- Metabolic rate (CMR*_glc_*): basal cerebral metabolic rate of glucose [Unit: μ *mol*/g/min].

**Figure 8:**
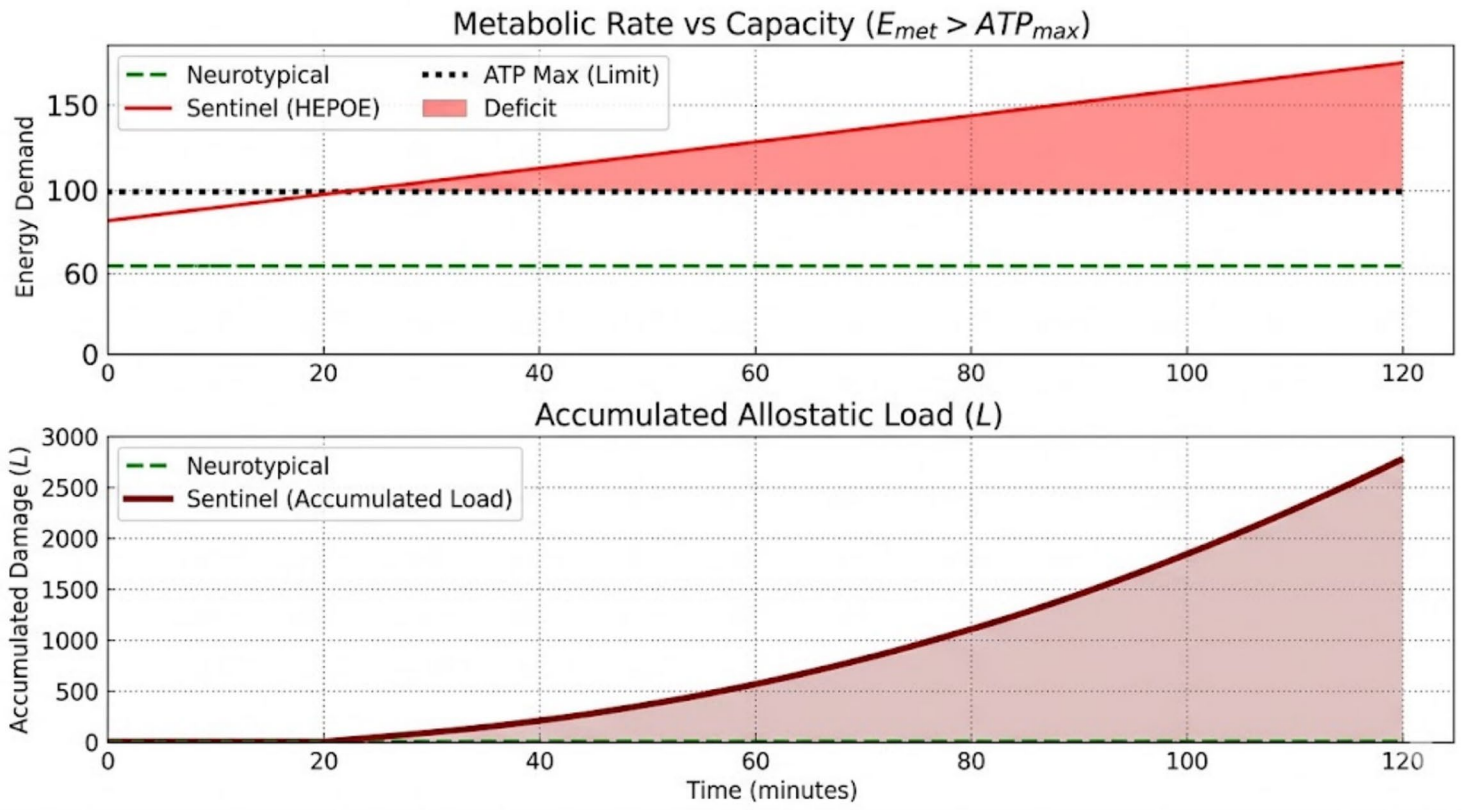
Temporal dynamics of Allostatic Load (*L*). Simulated comparison between phenotypes in a high-entropy environment. While the control phenotype (green) maintains metabolic stability (*E_met_ < ATP_max_*), the Sentinel Phenotype (red) exhibits rapid accumulation of bioenergetic debt, reaching the critical insolvency point (Breakpoint) at approximately 70 min of continuous exposure. Source: Brezolin and Freitas (2026).

Under high-entropy processing conditions (Sentinel Phenotype), the metabolic demand exceeds the basal rate. However, neuroenergetic literature (Brown and Ransom, 2007) suggests that glycogen acts as a critical buffer system, supplementing blood glucose to sustain axonal stability during acute demand. This physiological buffering extends the functional window to a range of 20 to 40 min before synaptic failure or severe local acidosis occurs. *In silico* agent-based modeling of this dynamic reveals a deterministic “Breakpoint” at approximately 70 min of continuous high-fidelity exposure. This corresponds to the transition from primary astrocytic glycogen depletion into anaerobic glycolysis, culminating in the neuroprotective downregulation clinically observed as “Brain Fog” (see Figure 8).

Consequently, the “70-min Breakpoint” observed in our computational simulation represents the cumulative sum of:

- Phase I (0–35 min): depletion of the primary astrocytic glycogen reserve (*T_acute_*).
- Phase II (35–70 min): the system enters a “dirty burn” state (anaerobic glycolysis), sustaining operation via the Lactate Shuttle until oxidative stress and acidosis trigger the neuroprotective downregulation mechanism (Adiaphoric Throttling or Brain Fog).

#### 7.3.4 Computational stress testing of the solvency inequality

The operational survival of the Sentinel hardware in stochastic environments is governed by the Predictive Solvency Inequality (*C ≥ H(E)* + Δ*P* + *W_erasure_*), as formally defined in Section 7.2.2.4.

**Figure 9:**
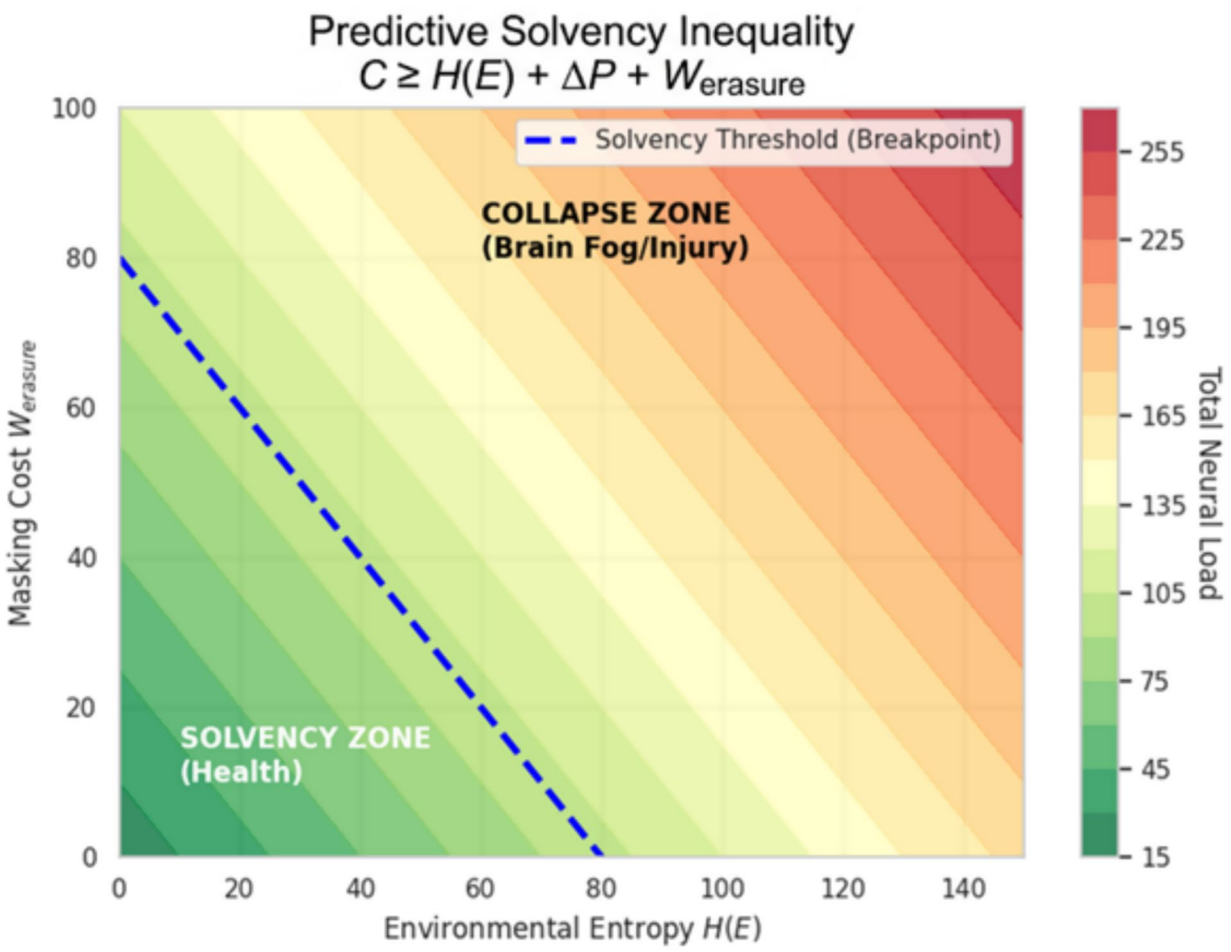
Predictive Solvency Map and Collapse Zones. Visualization of the inequality *C* ≥ *H(E)* + Δ*P* + *W_erasure_*. The dashed blue line demarcates the Neural Fidelity Limit (*C*). Regions above this line indicate metabolic insolvency (brain fog), demonstrating that Sentinel collapse is a deterministic function of environmental saturation and the demand for truth suppression (*W_erasure_*), independent of raw intellectual capacity. Source: Brezolin and Freitas (2026).

Computational stress testing of this inequality demonstrates that crossing the Solvency Threshold (*E_met_* > *ATP_max_*) makes systemic collapse a mathematical certainty, not a psychological variable. The Sentinel’s vulnerability is not a lack of high-fidelity capacity (*C*), but the exponential bioenergetic cost of maintaining predictive precision (Δ*P*) while actively managing erasure work (*W_erasure_*) in high-entropy environments (see Figure 9).

This is the most critical graph for clinical application. It intersects two variables: “Environmental Chaos” vs. “Effort to Disguise.”

- Green zone: where the Sentinel survives (honest environment, even if complex).
- Red zone: where Brain Fog occurs (dishonest and chaotic environment).

The blue line is the Solvency Frontier: crossing this line makes collapse mathematical, not psychological.

#### 7.3.5 Model limitations and temporal variability

This simulation assumes a constant high-entropy stream for the purpose of Stress Testing, aiming to identify the failure point within a ‘Worst-Case Scenario’. In real-world ecological scenarios, the presence of stochastic fluctuations and natural pauses allows for micro-windows of glycolytic recovery that are not accounted for in this algorithm.

Therefore, the “70-min Breakpoint” identified herein must be interpreted as the lower bound of solvency under maximum continuous load. Under intermittent load conditions or within environments of lower informational density, the window of functional operability tends to expand proportionally to the availability of bioenergetic recovery.

It is critical to emphasize that the “Solvency Frontier” is a dynamic threshold rather than a static limit. The exact position of this boundary is governed by the individual rate of ATP resynthesis and mitochondrial efficiency. This bioenergetic variability explains the metabolic differences observed among Sentinels: an individual with superior glycogen buffering capacity or higher antioxidant throughput will exhibit a more resilient solvency margin, effectively delaying the onset of ‘Brain Fog’ even under identical entropy loads (*H(E)*).

### 7.4 The dynamic solvency integral and systemic friction

While the Predictive Solvency Inequality establishes the static boundaries of neural fidelity, real-world cognitive performance is a time-dependent variable. To address the reviewer’s mandate for dynamic, testable frameworks, we expand the HEPOE architecture into a temporal model: the Brezolin Solvency Integral.

Furthermore, HEPOE is proposed as an etiologically agnostic Shell Theory (Meta-Architecture). Genetic polymorphisms, neuroinflammatory markers, or enzymatic imbalances do not invalidate the thermodynamic premise; instead, they function as variables of Systemic Friction (Ω), quantifying the biological resistance to energy resynthesis.

#### 7.4.1 The dynamic flux equation

The neurocognitive solvency (*S*) of the system at any given time (*t*) is mathematically defined as the cumulative balance between the rate of metabolic resynthesis and the total informational load, scaled by systemic friction:


$$S(t)=S_{0}+\int_{0}^{t}[\Phi (t)-(H{\left(E\right)}_{t}+\Delta P_{t}+W_{\text{erasure},t})\times \Omega ]\mathrm{dt}$$

Where:

- *S*(*t*) (Current Solvency Level): percentage of available operational “bandwidth” or battery at any given time [Unit: %].
- *S*_0_: Initial Solvency state at *t* = 0 (ideally 100% after restorative rest) [Unit: %].
- Φ(*t*) (Restoration Flux): the rate of astrocytic resynthesis and homeostatic restoration [Unit: %/min].
- (*H(E) +* Δ*P + W_erasure_*): the combined high-fidelity load, originally calculated in bits/min and normalized as a percentage of total systemic bandwidth depletion per minute to facilitate solvency and autonomous modeling [Unit: %/min].
- Ω (Systemic Friction/Impedance): the Systemic Friction and Impedance Coefficient that scales the load. In optimized hardware, Ω = 1.0. In systems with biochemical or genetic inefficiencies (e.g., enzymatic deficiencies or chronic inflammation), Ω > 1.0, causing an accelerated decay of *S(t)* [Unit: Dimensionless].
- *dt*: The time differential [Unit: min].
- Note on biological catalysts: factors that influence sensory precision and neural gain, such as neuromodulatory activity (e.g., the locus coeruleus-norepinephrine system), are hypothesized to act as biological catalysts within this framework. By artificially increasing the signal-to-noise ratio in high-fidelity states, these neuromodulators directly amplify the metabolic cost per processed bit, thereby acting as the physiological drivers that scale the Systemic Friction (Ω) and Neural Amplification (*N_amp_*) variables in our model.

In the Sentinel Phenotype, the intensive work of maintaining predictive precision (Δ*P*) and clearing informational noise (*W_erasure_*) frequently exceeds the maximum resynthesis rate (Φ), creating a deterministic *Negative Flux State* during high-fidelity tasks.

#### 7.4.2 Thermal self-preservation: brain fog as a circuit breaker

The transition from a state of high-resolution processing to the debilitating state known as “Brain Fog” has long been treated as a subjective psychological phenomenon. HEPOE Theory redefines this state as a mandatory Thermodynamic Safety Protocol. In High-Fidelity (Sentinel) architectures, the management of informational noise is not merely an energy-intensive task, but a thermal one.

According to Landauer’s Principle (Landauer, 1961), any logically irreversible manipulation of information, such as the erasure of a bit (*W_erasure_*), must result in the dissipation of a minimum amount of heat into the environment (*Q* ≥ *k_B_ T* ln 2) (Landauer, 1961). Further reviews emphasize that this thermodynamic limit is intrinsic to any computational process (Bennett, 1982). In the Sentinel Phenotype, where the volume of processed and subsequently “erased” data to maintain predictive precision (Δ*P*) is significantly higher than in neurotypical architectures, the cumulative heat dissipation becomes a non-trivial factor in neural homeostasis.

We propose that Brain Fog is a form of Neural Thermal Throttling. When the Brezolin Solvency Integral (*S*(*t*)) approaches a critical threshold, the system’s ability to manage the thermal byproducts of information processing fails. To prevent structural damage, the brain initiates a protective protocol that:

- Reduces predictive precision (Δ*P*): by lowering the resolution of sensory and cognitive processing, the system decreases the bit-rate, thus reducing heat generation.
- Diminishes erasure work (*W_erasure_*): by allowing informational noise to accumulate (perceived as mental confusion or sensory overload), the system avoids the thermodynamic cost of “cleaning” the synaptic workspace.

The ultimate goal of this “circuit breaker” is to prevent Glutamate-Mediated Excitotoxicity. The reuptake of glutamate from the synaptic cleft by astrocytes is an ATP-dependent process. In a state of Dynamic Insolvency (*S*(*t*) → 0):

- Energy levels are insufficient to maintain the high-speed recycling of neurotransmitters.
- The resulting accumulation of glutamate would lead to neuronal over-excitation, oxidative stress, and permanent structural damage (hardware failure).

Therefore, “Brain Fog” is not a failure of the mind, but a success of the hardware in preserving its physical integrity. By “dimming the lights” and “slowing the processor,” the Sentinel system avoids a catastrophic thermal meltdown. This protective state is a manifestation of allostatic load management under extreme informational demand (Sterling and Eyer, 1988). Following Laughlin’s principle of protection (Laughlin, 2001), the system triggers ‘Brain Fog’ as an emergent phase transition to prevent structural damage when metabolic solvency is compromised. By forcing a state of lower informational resolution, the hardware is protected from irreversible thermal and chemical degradation.

Figure 9 demonstrates the temporal application of the Brezolin Solvency Integral, simulating the “Time to Collapse”. It shows that, under the same environmental conditions, the Neurotypical maintains homeostasis (metabolic waste clearance equals production).

**Figure 10:**
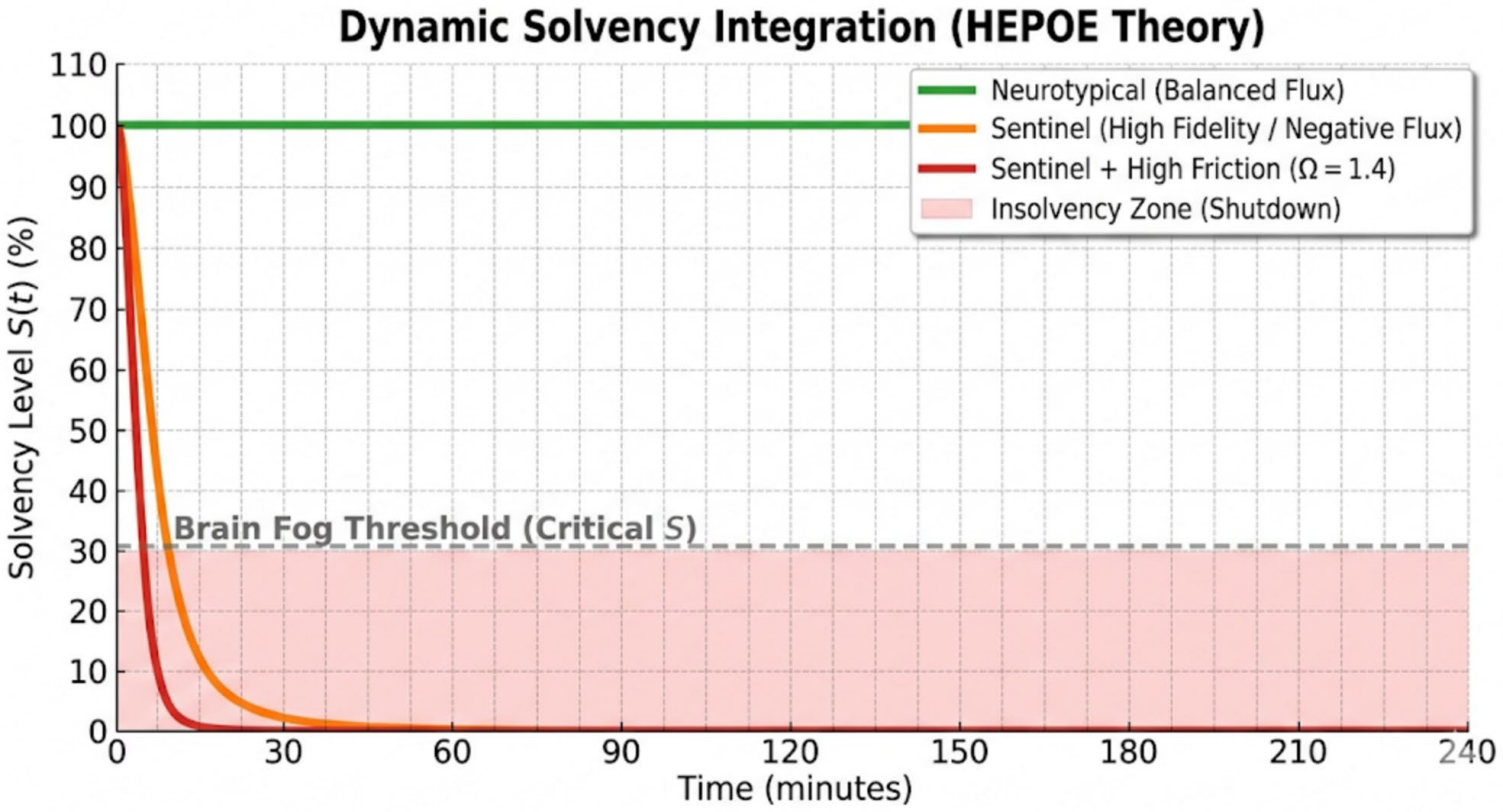
Dynamic Solvency Decay and The Breakpoint Phenomenon. Green Line (Neurotypical): represents a balanced energetic flux where resynthesis (Φ) is approximately equal to the operational load, allowing for sustainable long-term cognitive activity. *Orange Line (Sentinel)*: illustrates the “Negative Flux” characteristic of high-fidelity architectures. Due to high Δ*P* (precision) and *W_erasure_* (erasure work), solvency (*S*) decays steadily over time. *Red Line (High Friction Sentinel):* models a Sentinel with a High Systemic Friction Coefficient (Ω = 1.4). This represents individuals with biochemical inefficiencies (enzymatic deficiencies/inflammation), showing an accelerated trajectory toward insolvency. *Shaded Red Area (Insolvency Zone)*: the critical region below 30% solvency where the hardware is at risk of glutamate excitotoxicity. Source: Brezolin and Freitas (2026).

The Sentinel, however, due to high-fidelity processing, consumes its initial solvency (*S*_0_) at a rate that exceeds metabolic resynthesis, accumulating waste products (lactate/glutamate) faster than they can be cleared. The data indicates that the “Sentinel Breakpoint,” the moment of transition into Brain Fog, is not a random event but a mathematically predictable state. Furthermore, the inclusion of the Ω multiplier effectively compresses the operational window, explaining why individuals with identical cognitive loads but different biological friction levels reach exhaustion at significantly different rates. Ultimately, the shaded red area representing this metabolic insolvency becomes the physical manifestation of consolidated “Predictive Moral Injury” (see Figure 10).

#### 7.4.3 The recovery integral and residual insolvency

Restoration of the Initial Solvency (*S*_0_) is an active thermodynamic process defined by the Recovery Integral:


$$S_{\text{recovery}}=\int_{t_{\text{start}}}^{t_{\mathrm{end}}}[\eta \cdot \Phi (t)-H{\left(E\right)}_{\min}]\mathrm{dt}\;$$

Where:

- *S_recovery_*: the final recovered solvency level after a rest period [Unit: %].
- *η* (Eta): the Recovery Efficiency Coefficient, representing the individual’s metabolic capacity to convert rest into systemic solvency [Unit: Dimensionless].
- Φ(*t*): the rate of astrocytic resynthesis and homeostatic restoration [Unit: %/min].
- *H(E)_min_*: the residual environmental entropy during rest. If the environment is not “silent” (sensory-neutral), *H(E)* continues to drain the recovery flux [Unit: %/min].
- *t_start_*: the starting time of the rest or sensory isolation period [Unit: min].
- *t_end_*: the end time of the rest or sensory isolation period [Unit: min].
- *dt*: the time differential [Unit: min].

The HEPOE Shell Theory accounts for the high degree of variability observed in twice-exceptional (2e) individuals regarding sleep and isolation requirements:

- High-efficiency Sentinels (*η* · Φ ≫ 1): individuals who possess highly efficient clearing mechanisms. They can achieve *S*_0_ in shorter cycles (e.g., 4–6 h), provided the rest is of high quality.
- Low-efficiency Sentinels (*η* · Φ ≈ *H(E)_min_*): individuals with significant systemic friction (Ω), such as enzymatic deficiencies or neuroinflammation. For these, the recovery flux is nearly canceled out by residual noise, necessitating extended rest (8 h+) or total sensory isolation to achieve full re-solvency. These requirements reflect the physiological need to clear the ‘thermal debt’ accumulated during high-fidelity processing cycles.

If the rest period (*t*) is terminated before the integral reaches the value of *S*_0_, the system starts the next operational cycle with Residual Insolvency. This cumulative debt explains the “Crash” phenomenon: when *S(t)* begins at a suboptimal level day after day, the system reaches the Breakpoint (Brain Fog) increasingly earlier in the cycle. Without intervention (e.g., deep sensory “cooling”), this trajectory leads inevitably to chronic hardware failure, or Burnout.

**Figure 11:**
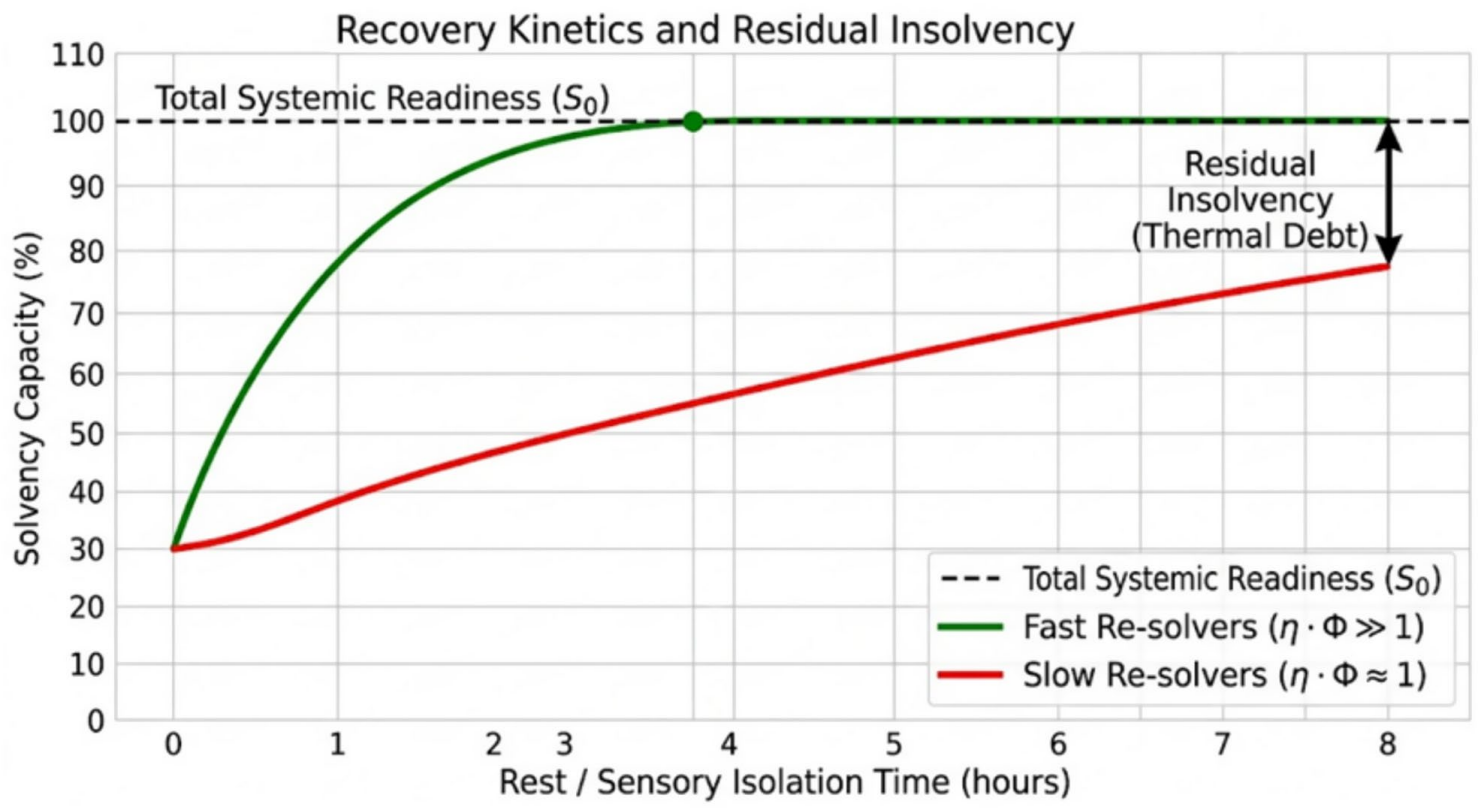
Recovery Kinetics and Residual Insolvency. Steep Curve *(η · Φ ≫ *1*)*. Represents “Fast Re-solvers” who possess high metabolic efficiency, reaching *S*_0_ within standard rest cycles. *Shallow Curve (η · Φ ≈ *1*)*. Represents “Slow Re-solvers” with high friction or low resynthesis rates, requiring extended isolation to clear the thermal debt. *Dashed Horizontal Line (S_0_)*. The baseline for total systemic readiness. Source: Brezolin and Freitas (2026).

This simulation validates the clinical necessity of Sensory Isolation and Thermodynamic Cooling as medical requirements for the Sentinel Phenotype. While recovery is a multi-factorial process, the data suggests that minimizing *H(E)_min_* (environmental noise) could be the most efficient catalyst to ensure that the recovery flux is fully directed toward resynthesis. By reducing the external entropic load, the system ceases the continued dissipation of informational heat, allowing the finite metabolic resources to prioritize the restoration of the neural hardware (see Figure 11).

## 8 Methodological framework

### 8.1 Research nature: theoretical synthesis and consilience

The present investigation is defined as Strategic Basic Research of a Theoretical-Conceptual nature, structured through the method of Rigorous Interdisciplinary Synthesis. Unlike isolated experimental studies that test discrete variables, this research adopts the approach of Consilience (Wilson, 1998) to integrate already validated biophysical laws (Biological Thermodynamics and the Free Energy Principle) with clinical phenomenology.

The choice not to conduct primary data collection at this stage is deliberate and methodologically grounded: current literature on Giftedness suffers from descriptive fragmentation, characterized by an abundance of behavioral data but an absence of a unified causal mechanism. Generating new clinical data without a prior bioenergetic framework would result only in further statistical noise.

Therefore, the validation of this study does not reside in the “discovery” of new empirical data, but in the formulation of a cohesive, testable theoretical model. It demonstrates the logical necessity between established biophysical premises (the neuronal ATP cost described by Laughlin, 2001) and observable behavior (described by Dabrowski, 1964). The HEPOE model thus operates as the necessary logical infrastructure, or causal map, that must precede and guide any future longitudinal experimental investigation.

The applied methodology is Theoretical Synthesis, utilizing the following pillars:

- Biological Thermodynamics: application of the laws of energy conservation and neuronal metabolic efficiency. The model’s validity proposes that ATP cost and oxidative stress act as physical constraints on processing. Although this study did not measure ATP directly, Neubauer and Fink (2009) demonstrated that high-intelligence brains exhibit differentiated neural efficiency, characterized by lower consumption in easy tasks and higher recruitment in complex tasks. The HEPOE Theory provides a causal infrastructure for these findings, hypothesizing that efficiency is not just a trait, but the direct result of free energy minimization. HEPOE interprets this phenomenon not merely as “efficiency,” but as a measurable biological datum of entropy management.
- Predictive Neuroscience: utilization of the Free Energy Principle (Friston, 2010) to explain brain function as a prediction engine that seeks to minimize entropy. HEPOE’s conceptual framework is established by demonstrating that the “Sentinel” is an optimized system for reducing environmental uncertainty through high-fidelity sampling.
- Interdisciplinary Systems Logic: the theory employs the rigor of systems analysis to reinterpret decades of clinical phenomenology (Dabrowski, Silverman, Renzulli). The methodological robustness is evaluated by its ability to unify all observed traits of giftedness under a cohesive set of biophysical principles, generating falsifiable hypotheses for future clinical trials.

### 8.2 Technical procedures: bibliographic and documentary research

The structural development of this theoretical-conceptual research was conducted as strategic basic research, grounded in a rigorous bibliographic survey. This methodological approach is essential for the construction of integrative models, as it allows for the synthesis of distinct but validated scientific domains into a unified framework:

- Search Strategy and Keywords: to ensure methodological traceability while honoring the interdisciplinary nature of this synthesis, the literature search was not conducted using a single restrictive Boolean intersection (which would yield null results given the novelty of this specific integration). Instead, we employed a Multi-Axial Search Strategy. Searches were conducted independently across three distinct domains to gather the foundational variables, using the following Boolean parameters:

1. Axis 1 (neuroenergetics & predictive processing): (“predictive coding” OR “active inference” OR “free energy principle”) AND (“neuroenergetics” OR “ATP resynthesis” OR “astrocytic glycogen” OR “thermodynamics”).
2. Axis 2 (phenomenology of high-fidelity): (“giftedness” OR “high cognitive ability” OR “twice-exceptional”) AND (“sensory gating” OR “latent inhibition” OR “working memory capacity”).
3. Axis 3 (systemic friction & clinical overlap): (“allostatic load” OR “oxidative stress” OR “burnout” OR “moral injury”) AND (“autism” OR “ADHD” OR “PTSD” OR “differential diagnosis”).

- Cross-disciplinary intersection: articles gathered from these independent axes were screened for empirical validity and then subjected to our theoretical synthesis protocol (detailed in Section 8.5). The intersection of these distinct domains, mapping the macroscopic traits of Axis 2 onto the bioenergetic constraints of Axis 1, constitutes the original intellectual contribution of the HEPOE framework.

#### 8.2.1 Document selection criteria

To ensure the replicability of this theoretical synthesis, the selection of literature followed strict criteria:

- Inclusion criteria: (1) Peer-reviewed articles and seminal academic texts published in internationally recognized scientific journals and academic presses; (2) Foundational research establishing the biophysical and neurocomputational limits of brain function (e.g., neural efficiency, the Free Energy Principle, and signal processing constraints); (3) Empirical research and classical literature detailing the phenomenology of High Ability/Giftedness and its clinical overlaps (ASD, ADHD, PTSD).
- Exclusion criteria: (1) Non-peer-reviewed theoretical opinion pieces lacking mechanistic or biological grounding; (2) Studies relying exclusively on psychometric data without neurobiological or systemic correlation; (3) Literature focused solely on pedagogical interventions without addressing the underlying cognitive hardware.

#### 8.2.2 Rules for weighting evidence and operational delimitation

To resolve theoretical conflicts, an integrative weighting rule was applied to bridge physical causality with clinical manifestation. Mechanistic weight was assigned to neurobiological constraints, thermodynamic limits of computation (e.g., Landauer’s Principle), and mathematical validations of the Free Energy Principle to establish the biological boundaries of the hardware. Concurrently, classical phenomenological literature (e.g., Dabrowski, Silverman) was assigned foundational clinical weight. These seminal works were utilized as the essential behavioral baseline, ensuring that the proposed biophysical mechanisms accurately mapped onto historically documented psychological outputs.

Furthermore, a strict translational protocol was applied for the operational delimitation of constructs. Subjective psychological categories were translated into objective systemic variables: “Giftedness” was strictly delimited as a Sentinel, a high-fidelity sampling hardware rather than an IQ score and “Overexcitability” was redefined as an open sensory gating mechanism. “Allostatic Load” was delimited as the quantifiable bioenergetic debt resulting from chronic prediction error and “Burnout” was operationally translated to systemic metabolic insolvency.

### 8.3 Data collection instruments and techniques

The data collection and bibliographic survey for the structuring of HEPOE Theory occurred continuously between May 2022 and January 2026. This period of approximately 44 months allowed for an exhaustive scan and the maturation of the interdisciplinary connections necessary for theoretical consilience.

The collection followed these rigor criteria:

- Systematic scanning: periodic consultations in high-impact databases (PubMed, Scopus, Web of Science, and Google Scholar);
- Evidence screening: selection of seminal works and systematic reviews addressing neuronal biophysics, ATP metabolism, and classical HA/G models;
- Data flow analysis: the long collection period allowed for the observation of the evolution of predictive neuroscience models (such as the Free Energy Principle), ensuring that HEPOE’s foundation was aligned with the area’s most recent discoveries.

The collection technique was Comparative Documentary Analysis. The instrument used was a systematic scan of high-credibility databases. Raw data on neuronal metabolic cost (ATP) and signal processing rates (gating) were analyzed, crossing them with the phenomenological descriptions of HA/G. Collection was exhaustive until theoretical saturation was reached, where new data no longer altered the fundamental structure of HEPOE Theory.

### 8.4 Operationalization, falsifiability, and demarcation of inferences

To transition from theoretical synthesis to empirical validation, the HEPOE framework explicitly demarcates its direct biophysical predictions from its broader macro-systemic implications.

#### 8.4.1 Core testable bioenergetic hypotheses

These constitute the foundational, biologically falsifiable claims of the model, generating testable hypotheses based on quantifiable biophysical variables:

- Metabolic debt hypothesis: under sustained high-entropy cognitive loads, individuals exhibiting the Sentinel Phenotype will demonstrate a significantly steeper trajectory of ATP depletion and metabolic byproduct accumulation (e.g., lactate) compared to neurotypical controls. This can be assessed through advanced neuroimaging techniques such as functional Magnetic Resonance Spectroscopy (fMRS).
- Gating and information flux: consistent with the “Open Gating” architecture, we hypothesize that the Sentinel hardware exhibits a reduced capacity for automated sensory filtering. This can be empirically tested through electrophysiological biomarkers of sensory gating and event-related potentials (ERPs), measuring the system’s objective permeability to irrelevant stimuli.
- The physical cost of masking: we propose that the active suppression of high-fidelity predictive models (social masking) results in a quantifiable increase in systemic allostatic load and localized thermal dissipation, as predicted by Landauer’s Principle. This provides a pathway for measuring the “compatibility tax” of neurodivergent adaptation through real-time physiological monitoring and autonomic signaling.
- Bayesian and structural equation modeling (SEM): to quantitatively validate the causal vectors of the HEPOE framework, future empirical protocols must integrate Bayesian modeling to track the dynamic updating of predictive priors (Free Energy Minimization) alongside Structural Equation Modeling (SEM). This will allow researchers to mathematically map the hierarchical relationship between systemic physical friction (Ω), the rate of ATP resynthesis, and the resulting accumulation of allostatic load.

#### 8.4.2 Macro-systemic inferences (future horizons)

While the biological substrate of the Sentinel hardware is physically quantifiable, the translation of these bioenergetic constraints into complex social dynamics constitutes a higher-order theoretical inference. The broad sociopolitical implications of Predictive Moral Injury, specific long-term identity formation, and large-scale public health policies proposed herein remain conceptual horizons. They require extensive future consilience between cellular thermodynamics, Bayesian modeling, and the social sciences before immediate clinical translation can be recommended.

### 8.5 Data analysis and treatment procedures

Data treatment followed a protocol of Integrative Theoretical Synthesis, applied under the rigor of Intelligent Systems Architecture.

- Coding phase: traits described in the literature (e.g., asynchrony, overexcitability) were coded as “Behavioral Outputs”;
- Inference phase: biological Thermodynamics logic was used to identify the “Root Cause” (Hardware Input) of these outputs;
- Synthesis phase: the final integration was processed using a systemic compatibility matrix, ensuring the proposed explanation for the “Sentinel” was isomorphic to signal processing models, mathematically coherent with the Free Energy Principle, and biologically sustainable by the neuronal energy cost. The HEPOE hardware operationalizes the fact that working memory capacity is a core predictor of general intelligence, often being perfectly correlated with Spearman’s g (Spearman, 2008; Kyllonen and Christal, 1990; Colom et al., 2004). This demonstrates that ‘general intelligence’ is the macroscopic manifestation of a hardware specialized in Free Energy Minimization.

It is concluded, therefore, that HEPOE Theory not only meets the requirements of methodological rigor but establishes a unit of knowledge where physics and phenomenology confirm each other. The consilience demonstrated here transforms giftedness from an observational category into a quantifiable biological fact, allowing science to move beyond the description of symptoms toward understanding the causality of the hardware.

### 8.6 Methodology and operational flow

Although HEPOE Theory offers a robust synthesis between biophysics and the phenomenology of High Abilities, it is imperative to delimit its current stage of development and the frontiers that still demand exploration:

- Theoretical-synthetic nature: the present phase of research is strictly theoretical and foundational. Although it utilizes neurobiological and thermodynamic data already independently validated, the HEPOE Theory is inherently falsifiable, aligning with the Popperian criterion for scientific demarcation (Popper, 1959). The model predicts that, under high-entropy cognitive loads, individuals identified as Sentinels will exhibit a significantly steeper ATP depletion curve and a higher accumulation of oxidative stress biomarkers compared to standard neurobiological baselines. The current absence of longitudinal data is thus framed not as a gap in evidence, but as the fundamental basis for hypothesis testing: the theory’s validity hinges on whether advanced bioenergetic monitoring confirms this differential metabolic cost during cognitive tasks.
- Experimental roadmap: the current model serves as the causal map that allows experimental neuroscience to test, for the first time, the direct correlation between Metabolic Cost and Predictive Moral Injury. By establishing these bioenergetic constraints, HEPOE provides the necessary framework to investigate how high-fidelity predictive hardware may lead to quantifiable somatic damage when operating in low-resolution environments.
- Gating measurability: currently, “high-fidelity sampling” is a logical deduction based on behavioral outputs and latent inhibition. The frontier of knowledge lies in the development of neuroimaging protocols or biomarkers that can quantify sensory gating in real-time under variable entropy loads. It must be understood that the Sentinel’s suffering is not a disproportionate emotional reaction, but the subproduct of error detection based on a high-fidelity statistical probability; the injury occurs because the hardware “reads” the collapse before it becomes obvious to the system.
- Epigenetic variability: the theory focuses on the hardware architecture (“The Sentinel”), but recognizes that its expression is modulated by epigenetic and environmental factors not yet detailed in this initial model.
- Clinical scalability: as a proposed paradigm shift, the application of HEPOE in differential diagnosis (ASD/ADHD) requires the creation of specific screening instruments that translate “hardware logic” into clinical practice, which exceeds the scope of this paper.

Furthermore, the theory highlights the vital necessity of “Recovery Windows” (informational silence) for ATP resynthesis and stabilization of neural error-detection (Bechtereva, 1978), mitigating the cumulative allostatic load (Sterling and Eyer, 1988) and restoring predictive homeostasis, which scientifically validates the Sentinel’s inherent biological need for periodic isolation.

Therefore, HEPOE Theory establishes the necessary conceptual framework for the next generation of neurobiological research to stop treating giftedness as a statistical deviation and begin investigating it as a phenomenon of biological engineering.

The limitations outlined here are not obstacles, but invitations for the experimental validation of a new paradigm that prioritizes processing logic over behavioral observation.

In this context, future research should explore how the Sentinel manages “wicked problems” (Rittel and Webber, 1973) and the long-term bioenergetic impact of chronic boredom (Götz et al., 2014). Specifically, it is crucial to investigate how these high-entropy inputs, or the lack thereof, affect the stabilization of the predictive model over time.

Furthermore, clinical research should investigate the “HEPOE Breakpoint,” defined as the metabolic threshold where the computational cost of entropy reduction exceeds the rate of mitochondrial ATP resynthesis. We hypothesize that the phenomenon commonly described as “Brain Fog” serves as a neuroprotective thermal downregulation mechanism, triggered to prevent systemic oxidative damage when the bioenergetic gradient cannot be maintained.

Ultimately, the visualization proposed in Figure 1, reinforced by the operational flow in Table 6, demystifies the phenomenon of the Sentinel. It demonstrates that the observed systemic fragility (injury) is directly proportional to the predictive power (output). Thus, future experimental validation should not seek to ‘fix’ the hardware, but to measure its thermodynamic efficiency and provide the necessary environmental buffering. This shift from clinical correction to bioenergetic support is the fundamental legacy of the HEPOE framework.

**Table 6:** Operational flow of the Sentinel hardware (HEPOE).

Process stage	Hardware mechanism (biophysics)	Systemic outcome (phenomenology)
1. Input	Open sensory gating and reduced latent inhibition (Carson et al., 2003).	Massive sampling of raw data and hypersensitivity.
2. Throughput (processing)	P-FIT network efficiency (Jung and Haier, 2007) and search for isomorphies (Universal Link).	Complex pattern recognition and leap learning (Rogers, 1986).
3. Constraint (cost)	Accelerated ATP resynthesis (Laughlin, 2001) and high Allostatic Load (Sterling and Eyer, 1988), necessitating informational silence (Bechtereva, 1978) and synaptic homeostasis (Tononi, 2009).	Metabolic exhaustion, periodic isolation, and regulatory sleep.
4. Output	Free Energy Minimization (Friston, 2010) and high-fidelity prediction.	Predictive Moral Injury and anticipatory ethical signaling.

Source: Brezolin and Freitas (2026), based on the principles of Isomorphy and Predictive Processing.

## 9 Discussion: systemic and clinical implications

### 9.1 From pathology to bioenergetics: diagnostic and public health revisions

The HEPOE framework posits that the current overlap between the Sentinel Phenotype and clinical diagnoses (e.g., ASD, ADHD, Bipolar Disorder) reflects what can be viewed as a systemic failure in public health. We argue that the “symptoms” often medicated represent the metabolic cost of a high-performance engine running without “fuel” (meaning) or proper cooling (isolation).

Beyond ATP constraints, it is hypothesized that the accumulation of metabolic by-products, such as lactate signaling and glutamate-induced oxidative stress, constitutes the primary “noise” in high-performance engines. Future investigations should seek to offer a bioenergetic alternative to symptom suppression, focusing on maintaining “bioenergetic solvency.” Therefore, we propose the creation of a “Non-Pathological Neurodivergent Status” in future revisions of international diagnostic manuals (WHO/ICD). This status aims to protect Sentinels from iatrogenic errors, shifting clinical focus from correcting a “disorder” to managing the specific allostatic load of high-fidelity hardware.

### 9.2 The right to entropy: cognitive accessibility in education

High-entropy processing is not a preference but a survival mechanism for the Sentinel hardware. Educational systems based on linear repetition and slow pacing are hypothesized to induce a state of “hypo-stimulation,” leading to neurobiological collapse and systemic burnout. We introduce the concept of Cognitive Accessibility: the legal right to environments that match the individual’s processing density. Just as physical accessibility is guaranteed by law, identifying “Homeostatic Recovery Windows” and informational density as fundamental neurobiological rights may prevent “informational starvation” and validate the Sentinel’s need for complexity as a requirement for health, not just a luxury.

### 9.3 Public policy and human rights

Recognizing the Sentinel as a biological specialization provides a strong rationale for corresponding legal protection. Public policies would benefit from transitioning from viewing this population as a “privileged elite” to identifying them as a vulnerable biological minority prone to Predictive Moral Injury. From a public health perspective, providing the environmental buffering necessary is crucial to convert this high potential into innovation rather than trauma, protecting them from the somatic damage caused by the continuous signaling of systemic errors that the environment refuses to acknowledge.

### 9.4 Biomimetics and AI: solving wicked problems

From a computational standpoint, the HEPOE algorithm, modeled on the Sentinel’s ability to optimize high-entropy systems, offers a theoretical biomimetic roadmap for next-generation Artificial Intelligence. By mimicking the biological hardware designed for entropy reduction, AI architectures could potentially address Wicked Problems (Rittel and Webber, 1973) with unprecedented energy efficiency, establishing a bridge between biological consilience and machine intelligence.

### 9.5 Model limitations and cautious positioning

While the HEPOE Theory provides a robust bioenergetic and thermodynamic foundation for understanding the Sentinel Phenotype, it is imperative to strictly define the boundaries of its current applicability.

We explicitly clarify that the present work constitutes a theoretical and hypothesis-driven framework. At this stage of development, the model has not yet been empirically validated through targeted, *in vivo* neuroenergetic tracking or longitudinal clinical trials specific to the parameters defined herein. Consequently, its primary contribution remains heuristic and conceptual.

The HEPOE framework is rigorously designed to generate multidisciplinary discussion, reframe the epistemological approach to high-fidelity cognitive architectures, and guide future empirical research. It does not offer, nor does it currently intend to serve as, an operational or clinically applicable diagnostic tool. Direct translations of this predictive model into clinical reasoning, therapeutic interventions, or public policy considerations must be preceded by comprehensive experimental testing to prevent any overinterpretation of its current level of evidential support.

## 10 Final considerations on the thermodynamic question

### 10.1 The paradigm shift: the symptom as a defense protocol

The application of the HEPOE analysis (Brezolin and Freitas, 2026) proposes a biophysical recontextualization of the 2e clinical practice. The observed acute manifestations, such as Meltdown and Shutdown, are understood here beyond their traditional symptomatological classification. From this engineering perspective, these events do not represent “behavioral failures” but rather systemic saturation protocols (Thermodynamic Protection Mechanisms).

They are modeled to indicate the exact moment when the High-Fidelity system, in an attempt to preserve its structural integrity, reaches energetic insolvency in the face of the critical convergence between high predictive demand (Δ*P*) and total sensory permeability (*H(E)*). Therefore, treating the behavioral consequence without addressing the thermodynamic cause results in an incomplete intervention.

### 10.2 Thermodynamic modulation strategies and entropy mitigation

The understanding of the “Knowledge of Collapse” as an output of a high-fidelity Early Warning System (EWS) radically alters the therapeutic approach. Instead of treating the anticipation of exhaustion as an anxiety symptom, the focus must shift toward Bioenergetic Solvency Management.

Therapeutic intervention for the 2e Sentinel Phenotype must move beyond the “Error Correction” model, adopting a “Resource Management” model as the foundation for sustainability:

- Pillar 1: preventive entropy reduction (shielding the neural architecture): since the High-Fidelity regime prevents automatic noise filtering (Open Gating), ATP preservation requires an exogenous reduction of the data load. The use of Active Noise Cancellation (ANC) and light spectrum control should not be viewed as mere comfort or isolation, but as Personal Protective Equipment (PPE) necessary to reduce the basal load and free up FLOPS (floating-point operations per second) for higher cognition. It is not avoidance, but load balancing to maintain solvency. Specifically, the implementation of ANC technologies acts as a direct intervention in the reduction of the *H(E)* variable (Environmental Entropy) within the predictive solvency inequality. By filtering stochastic acoustic noise, the system prevents the ‘Encoding Imperative’ from triggering non-convergent computational cycles. This exogenous entropy reduction effectively spares ATP resources, reallocating the metabolic budget toward high-fidelity higher cognition and delaying the onset of systemic insolvency. For an Open Gating system, the exogenous reduction of irrelevant bits is the only way to preserve the ATP budget for superior cortical functions.
- Pillar 2: battery monitoring (bio-interoception): the patient must be trained in High-Precision Interoception to recognize the subtle signs of increasing thermodynamic cost, acting as the “background noise” of interoceptive vigilance. Early recognition of the prediction error rate allows for interventions and “Sensory Silence” breaks (buffer resets) to dissipate residual entropy before the system reaches the Landauer limit (Landauer, 1961) and triggers the thermal Shutdown protocol.
- Pillar 3: validation of Predictive Moral Injury (reduction of Δ*P*): therapy should aim to validate the physical suffering caused by meta-prediction. It is imperative to note that ‘Moral Injury’ here is not a metaphorical construct. Neuroimaging literature (e.g., Eisenberger et al., 2003) establishes that social and ethical dissonance activates the same neural substrates as physical pain (Anterior Cingulate Cortex). Therefore, the ‘Predictive Moral Injury’ described in HEPOE is conceptualized as a literal physiological event, specifically a glutamatergic storm triggered by the detection of systemic error, grounding the concept firmly in somatic reality rather than sociology. Knowing that the system will fail (EWS) while the environment ignores the signals generates secondary oxidative stress through an infinite Inference Loop. The conflict between high-fidelity prediction (knowing failure is imminent) and external invalidation (the environment stating “everything is fine”) creates a Data Parity Conflict, exponentially raising processing costs. Reconciling internal calculation with external reality is vital to cease the expenditure of Free Energy and avoid cumulative trauma. This halts the reprocessing loop and reduces the cost of free energy (Friston, 2010).

### 10.3 The thermodynamic cost of adaptive neuroplasticity

It is fundamental to qualify the adaptive capacity of the nervous system from the perspective of energy conservation. Although neuroplasticity is a potent biological resource, the HEPOE Theory postulates that it is not thermodynamically neutral; every structural adaptation carries a metabolic implementation cost. Adapting a High-Fidelity Architecture (Sentinel) to endure a low-resolution or sensorially toxic environment consumes resources (ATP and buffering capacity) that, teleologically, should be allocated toward the development of higher-order cognitive functions.

Therefore, interventions focused exclusively on exposure desensitization (“training” noise endurance) face a bioenergetic trade-off: the “battery” of plasticity is spent on ensuring homeostatic maintenance, reducing the available budget for systemic evolution. The result is not a cost-free adaptation, but a neural architecture operating under a “Compatibility Tax,” diverting processing efficiency toward survival management.

### 10.4 Disclaimer on emergent complexity

Although this model is applied here to the 2e phenotype, the HEPOE equation (*C ≥ H(E) +* Δ*P + W_erasure_*) should be understood as a fundamental thermodynamic law of cognition. It defines the universal boundary conditions for any high-fidelity information processing system, biological or artificial, where structural integrity is a function of energetic solvency. The numerical stability and stress testing of this inequality are formally validated in Figure 8, which maps the precise coordinates of the Predictive Solvency Threshold.

This mathematical boundary, however, is not a static ceiling but a dynamic frontier where biology meets phenomenology. While the HEPOE Theory utilizes information physics and thermodynamics as the “fundamental ruler” to map the origin of exhaustion in the 2e phenotype, we recognize that the simplicity of the physical laws described herein flows into a vast and multifaceted phenomenological complexity.

The exhaustion of metabolic resources and the saturation of information channels generate external implications, including psychological, social, emotional, and pedagogical factors, that transcend the scope of this initial model. The “energetic insolvency” we define at the cellular level propagates through the system, manifesting as the fragmented narratives of trauma or the exhaustion of social shielding.

Mapping how this “energetic insolvency” translates into trauma dynamics, identity formation, and learning barriers is an ongoing task. This task must integrate the physics proposed here with the human and social sciences, recognizing that while physics may be a single-line rule, what it generates is the entire universe.

### 10.5 Future directions: systemic and clinical implications of the HEPOE model

The bioenergetic framework proposed herein opens critical avenues for interdisciplinary research. Specifically, the high metabolic cost of the Sentinel Phenotype suggests systemic trade-offs that warrant investigation. However, we emphasize that while the mechanism of high-fidelity predictive processing is intrinsic to these neurocognitive architectures, the clinical outcome is not uniform. The progression from high metabolic consumption to systemic pathology (such as immune dysfunction or burnout) is likely modulated by a multivariate set of factors, including genetic, epigenetic, and environmental variables, that determine the individual’s “Thermodynamic Resilience.”

Therefore, we posit that generic clinical approaches are insufficient. Future research must focus on identifying specific biomarkers and stress thresholds that influence this bioenergetic system. As outlined in our discussion on Sentinel Levels, the impact of high-fidelity processing varies significantly based on individual constraints and environmental support. We hypothesize that chronic energy allocation to hypervigilant neural networks may deplete resources from peripheral systems, but the threshold for this depletion requires precise quantification through further clinical studies. This would help clarify why some individuals maintain homeostatic balance while others experience immune hyper-reactivity (allergies) or autoimmune disorders (immune system brownout).

Future works will also explore the mechanics of trauma as neural hysteresis (plastic deformation of predictive models) and the metabolic cost of high-fidelity social simulation (empathic fatigue). These extensions aim to transition the field from purely psychological interventions to bio-physically grounded management strategies, prioritizing the identification of the specific bio-energetic limiters that govern health outcomes in this population.

Ultimately, this work lays the foundation for a broader epistemological shift: the integration of Materials Science, Solid-State Physics, and Thermodynamics into General Medicine. We propose that the human body must be analyzed as a physical system subject to entropy, stress–strain curves, and material fatigue. The application of the HEPOE framework extends beyond diagnostic modeling; it offers a profound clinical paradigm shift for Sentinel Phenotypes, including the twice-exceptional (2e) population. By mathematically demonstrating that traits such as the Imposter Phenomenon, paralyzing perfectionism, and justice sensitivity are not inherent psychological deficits, but rather entropic byproducts of high-fidelity predictive processing, this model actively depathologizes the Sentinel experience. It shifts the therapeutic focus away from correcting perceived emotional flaws, redirecting it toward managing the real thermodynamic and computational costs (*W_erasure_*) of navigating low-resolution environments with high-resolution neural architectures.

Future research should quantify properties such as neural impedance matching, systemic resonance, and percolation thresholds to explain phenomena like ‘meltdowns’ or cognitive viscosity. This transdisciplinary approach aims to establish a new paradigm for precision medicine, where health could be measured by the individualized assessment of thermodynamic efficiency and physical compliance.

Furthermore, future research should explicitly correlate the Systemic Friction Coefficient (Ω) with specific genetic polymorphisms, such as those linked to mitochondrial efficiency and OXPHOS complex variations (Wallace, 2005), or the Astrocyte-Neuron Lactate Shuttle (Magistretti and Allaman, 2015). This transdisciplinary approach aims to successfully close the loop between genetics and macro-thermodynamics, explaining why Sentinels under identical informational loads exhibit vastly different rates of bioenergetic decay.

### 10.6 Final considerations: the ethics of high-fidelity

Twice-Exceptionality is not a paradox of competence, but an Efficiency Dilemma. The Sentinel Phenotype possesses an extraordinary processing fidelity, but it operates with an equally extraordinary fuel consumption. Bioethical justice for these individuals does not lie in forcing them toward statistical normality (neurotypicity), but in providing the energetic infrastructure necessary for their High-Fidelity to operate within thermal safety limits, without destroying the structural biological substrate that sustains it.

Recognizing the “Knowledge of Collapse” not as a software error, but as a high-precision Early Warning System (EWS), is what ensures the integrity of the structural biological substrate. Understanding the physics behind the suffering is the first step toward transforming vulnerability into sustainability and collapse into potency.

## 11 Conclusion

The HEPOE Theory presented herein does not merely offer a new definition of Giftedness; it proposes a theoretical framework for the thermodynamic emergence of the Sentinel Phenotype. By hypothesizing that high-fidelity is a function of hardware specialized in entropy reduction, this treatise challenges the outdated dichotomy between “gift” and “disorder.” Consequently, the acceptance of this bioenergetic reality suggests the need for significant shifts in three fundamental pillars of society: Education, Public Policy, and Human Rights.

Ultimately, this treatise serves as the foundational architecture for a research roadmap spanning Doctoral and Post-Doctoral investigations. By uniting the Laws of Thermodynamics with Clinical Psychology, the HEPOE paradigm strives to achieve the long-sought Consilience (Wilson, 1998), defined as the unity of knowledge.

We conclude that the clinical exhaustion and ‘Predictive Moral Injury’ observed in these individuals are conceptualized not as inherent flaws, but as a situational pathology emerging when a high-fidelity system is forced to operate in low-resolution or ethically incoherent environments.

We propose that the Sentinel functions not as an anomaly, but as a specialized evolutionary adaptation capable of navigating chaos. The suffering observed is not a defect, but the cost of seeing the truth before the rest of the system. The mission of clinical science, henceforth, should expand beyond attempting to “cure” the Sentinel, aiming instead to foster environments capable of enduring the precision of its vision.

## Data Availability

The computational model was developed using Python 3.10, NumPy, and Matplotlib libraries, which established the foundational validation module for the HEPOE Algorithm architecture. To ensure absolute reproducibility, methodological rigor, and computational transparency, the complete source code utilized to generate the synthetic datasets and in silico stochastic simulations is provided directly within Supplementary Data Sheet 1. Furthermore, the clinical scaling parameters and metabolic biomarkers associated with the Systemic Friction Coefficient (Ω) are detailed in Supplementary Data Sheet 2.

## Author’s note

This manuscript is an original theoretical synthesis developed by the authors. We explicitly declare that the lead author is visually impaired and utilizes assistive technologies, including screen readers and specialized text-processing software, for the production, editing, and formatting of this work. While these digital tools facilitate accessibility and language refinement, the conceptual framework (HEPOE Theory), the “Sentinel Phenotype” ontology, all mathematical modeling, and every scientific conclusion presented herein are the exclusive result of the authors’ original intellectual research and scientific syntheses. Camila L. M. Brezolin and Sidnei B. Freitas*: Authors of the HEPOE Theory.*
