# Supplementary material for: The Sentinel Phenotype: a theoretical bioenergetic and neurobiological framework for high-fidelity predictive systems (HEPOE Theory)
Source: Front Neurosci. 2026 May 29;20:1785088. doi: 10.3389/fnins.2026.1785088 (PMC13260446; doi:10.3389/fnins.2026.1785088)
Supplement: Supplementary file 1 [file Data_Sheet_1.docx]

**DATA SHEET 1: SOURCE CODE FOR COMPUTATIONAL VALIDATION**

**Figure 6:**

import numpy as np

import matplotlib.pyplot as plt

import seaborn as sns

def generate_brain_fog_figure():

# Visual style consistent with HEPOE Theory

sns.set_theme(style="whitegrid")

plt.style.use('default')

# Time and Solvency Parameters

T_max = 240

time = np.linspace(0, T_max, T_max)

S0 = 100

# Decay rate mathematically adjusted to hit 30% at 72 minutes

# (100 - 30) / 72 = drop of approx 0.972% per minute

change_per_min = -0.9722

# Applying the Dynamic Solvency Integral

S = np.zeros(T_max)

S[0] = S0

for t in range(1, T_max):

S[t] = max(0, S[t-1] + change_per_min) # Locks at Zero (Shutdown)

# Chart Configuration

plt.figure(figsize=(10, 6))

# Linear Decay Curve (The Real Math)

plt.plot(time, S, color='#d62728', linewidth=3, label='Sentinel Phenotype (High Friction)')

# Brain Fog Line

plt.axhline(y=30, color='gray', linestyle='--', linewidth=2, label='Brain Fog Threshold (30%)')

plt.fill_between(time, 0, 30, color='gray', alpha=0.2)

# Arrow and Surgical Annotation

plt.annotate('Enters Brain Fog\n~72 min', xy=(72, 30), xytext=(90, 45),

arrowprops=dict(facecolor='black', shrink=0.05, width=2, headwidth=8),

fontsize=11, fontweight='bold')

# Titles and Labels

plt.title('HEPOE Dynamic Solvency Validation: Sentinel Phenotype', fontsize=14, fontweight='bold', pad=15)

plt.xlabel('Time (minutes)', fontsize=12)

plt.ylabel('Solvency Capacity (%)', fontsize=12)

# Limits

plt.xlim(0, 240)

plt.ylim(0, 105)

# Legend

plt.legend(loc='upper right', frameon=True, shadow=True, fontsize=11)

plt.tight_layout()

plt.savefig('Figure_BrainFog_Onset.png', dpi=300)

plt.show()

if __name__ == "__main__":

generate_brain_fog_figure()

**Figure 7:**

import numpy as np

import matplotlib.pyplot as plt

import seaborn as sns

# Style Configuration

plt.style.use('default')

sns.set_theme(style="whitegrid")

# --- GRAPH 2: LANDAUER'S PRINCIPLE (W_erasure) ---

def plot_landauer():

bits_erased = np.arange(0, 1000, 1)

# Fundamental Physical Constants

kB = 1.38e-23

T = 310 # 310K (37°C - Body Temperature)

# NEURAL AMPLIFICATION COEFFICIENT (N_amp)

# Biophysical Justification for Dissipation Scale:

# Landauer's Limit (kBTln2) applies to 1 microscopic bit of freedom.

# However, suppressing a cognitive 'bit' (insight/masking) in the Sentinel Phenotype

# is an emergent macroscopic event. The factor 1e20 derives from aggregating:

# 1. Thermodynamic Inefficiency: The brain operates > 10^6 above Landauer's limit (Laughlin, 2001).

# 2. Synaptic Density: The P-FIT network recruits ~10^9 synapses/second for active inhibition.

# 3. Molar Scale: Conversion of quantum events to ATP hydrolysis (Avogadro Scale). N_amp = 1e20

# Calculation of Total Dissipated Energy (E)

# E = (Erased Bits * Unit Landauer Cost) * Systemic Amplification

E = bits_erased * (kB * T * np.log(2)) * N_amp

plt.figure(figsize=(8, 5))

plt.plot(bits_erased, E, color='red', linewidth=2.5)

# CORRECTION: Changed 'Formula 2' to 'Figure 6' to match PDF

plt.title('Landauer\'s Principle (Erasure Cost)\n*Q ≥ k_B_ T ln 2*', fontsize=14)

plt.xlabel('Erased Bits (Masking)', fontsize=12)

plt.ylabel('Amplified Energy Cost (Arbitrary Units)', fontsize=12)

plt.fill_between(bits_erased, E, color='red', alpha=0.1)

plt.grid(True, linestyle=':', alpha=0.6)

plt.tight_layout()

plt.savefig('Figure_S2_Landauer.png')

plt.close()

**Figure 8:**

import numpy as np

import matplotlib.pyplot as plt

import seaborn as sns

# Style Configuration

plt.style.use('default')

sns.set_theme(style="whitegrid")

# --- GRAPH 3: ALLOSTATIC LOAD INTEGRAL (L) ---

def plot_allostatic_load():

time = np.arange(0, 120, 1) # 120 minutes

atp_max = 100

# Neurotypical: Low constant cost

e_met_nt = np.full_like(time, 60)

# Sentinel: High and increasing cost (Spinning in Place)

e_met_st = 90 + (time * 0.5)

# Integral Calculation L (Accumulated)

load_nt = np.cumsum(np.maximum(0, e_met_nt - atp_max))

load_st = np.cumsum(np.maximum(0, e_met_st - atp_max))

fig, (ax1, ax2) = plt.subplots(2, 1, figsize=(10, 8), sharex=True)

# Subplot 1: Instantaneous Metabolic Rate

ax1.plot(time, e_met_nt, label='Neurotypical', color='green', linestyle='--')

ax1.plot(time, e_met_st, label='Sentinel (HEPOE)', color='red')

ax1.axhline(y=atp_max, color='black', linestyle=':', linewidth=2, label='ATP Max (Limit)')

# CORRECTION: Changed 'Formula 3a' to 'Figure 7' to match PDF

ax1.set_title('Metabolic Rate vs Capacity (*E_met_* > *ATP_max_*)', fontsize=12)

ax1.set_ylabel('Energy Demand', fontsize=10)

ax1.legend()

ax1.fill_between(time, e_met_st, atp_max, where=(e_met_st > atp_max), color='red', alpha=0.2, label='Deficit')

# Subplot 2: Accumulated Allostatic Load

ax2.plot(time, load_nt, label='Neurotypical', color='green', linestyle='--')

ax2.plot(time, load_st, label='Sentinel (Accumulated Load)', color='darkred', linewidth=2.5)

# CORRECTION: Changed 'Formula 3b' to 'Figure 7' to match PDF

ax2.set_title('Accumulated Allostatic Load (*L*)', fontsize=12)

ax2.set_xlabel('Time (minutes)', fontsize=10)

ax2.set_ylabel('Accumulated Damage (L)', fontsize=10)

ax2.fill_between(time, load_st, color='darkred', alpha=0.1)

ax2.grid(True, linestyle=':', alpha=0.6)

plt.tight_layout()

plt.savefig('Figure_S3_Allostatic_Load.png')

plt.close()

**Figure 9:**

import numpy as np

import matplotlib.pyplot as plt

import seaborn as sns

# Style Configuration

plt.style.use('default')

sns.set_theme(style="whitegrid")

# --- GRAPH 4: PREDICTIVE SOLVENCY INEQUALITY ---

def plot_solvency():

# C >= H(E) + dP + W

H = np.linspace(0, 150, 100) # Environmental Entropy

W = np.linspace(0, 100, 100) # Erasure Work (Masking)

H_grid, W_grid = np.meshgrid(H, W)

dP = 20 # Fixed Predictive Precision

C_neural = 100 # Fixed Neural Capacity

# Total Load

Load = H_grid + dP + W_grid

# Visualization

plt.figure(figsize=(8, 6))

contour = plt.contourf(H_grid, W_grid, Load, levels=20, cmap='RdYlGn_r', alpha=0.8)

cbar = plt.colorbar(contour)

cbar.set_label('Total Neural Load', rotation=270, labelpad=15)

# Solvency Threshold Line (Breakpoint)

# W = C - dP - H

limit_line = C_neural - dP - H

plt.plot(H, limit_line, color='blue', linewidth=3, linestyle='--', label='Solvency Threshold (Breakpoint)')

# CORRECTION: Changed 'Formula 4' to 'Figure S4' to match PDF

plt.title('Predictive Solvency Inequality\n*C ≥ H(E) + ΔP + W_erasure_*', fontsize=14)

plt.xlabel('Environmental Entropy H(E)', fontsize=12)

plt.ylabel('Masking Cost *W_erasure_*', fontsize=12)

plt.legend(loc='upper right')

plt.text(10, 10, 'SOLVENCY ZONE\n(Health)', color='white', fontweight='bold', fontsize=12)

plt.text(60, 80, 'COLLAPSE ZONE\n(Brain Fog/Injury)', color='black', fontweight='bold', fontsize=12)

plt.ylim(0, 100)

plt.xlim(0, 150)

plt.tight_layout()

plt.savefig('Figure_S4_Solvency.png')

plt.close()

# Run all functions

if __name__ == "__main__":

plot_shannon()

plot_landauer()

plot_allostatic_load()

plot_solvency()

**Figure 10:**

import numpy as np

import matplotlib.pyplot as plt

# --- Parameters Based on HEPOE Theory ---

S0 = 100 # Initial Solvency (Capacity in %)

T_max = 240 # Observation time (240 minutes / 4 hours)

time = np.linspace(0, T_max, T_max)

# Profile 1: Neurotypical (Balanced Flux)

# Low resolution, efficient noise filtering

H1, dP1, W1, phi1, omega1 = 10, 5, 5, 20, 1.0

load1 = (H1 + dP1 + W1) * omega1

# Profile 2: Sentinel Phenotype (Negative Flux)

# High fidelity (dP), high erasure cost (W)

H2, dP2, W2, phi2, omega2 = 25, 20, 15, 25, 1.0

load2 = (H2 + dP2 + W2) * omega2

# Profile 3: Sentinel with High Systemic Friction (e.g., Inflammation/ enzymatic deficiencies)

# Same load as Sentinel, but with a high Omega coefficient

H3, dP3, W3, phi3, omega3 = 25, 20, 15, 25, 1.4

load3 = (H3 + dP3 + W3) * omega3

# --- Solvency Integration Function ---

def calculate_solvency(S0, phi, load, T):

"""

Calculates the solvency S(t) over time based on the rate of resynthesis (phi)

and the total metabolic load.

"""

S = np.zeros(T)

S[0] = S0

for t in range(1, T):

# S(t) = S(t-1) + (Resynthesis Rate - Total Load)

# Where Total Load = (H + dP + W) * Omega

change = phi - load

S[t] = max(0, S[t-1] + change) # Solvency does not drop below zero (Shutdown)

return S

# Simulation Execution

S1 = calculate_solvency(S0, phi1, load1, T_max)

S2 = calculate_solvency(S0, phi2, load2, T_max)

S3 = calculate_solvency(S0, phi3, load3, T_max)

# --- Data Visualization ---

plt.figure(figsize=(12, 7))

# Plotting the curves

plt.plot(time, S1, label='Neurotypical (Balanced Flux)', color='#2ca02c', linewidth=3)

plt.plot(time, S2, label='Sentinel (High Fidelity / Negative Flux)', color='#ff7f0e', linewidth=3)

plt.plot(time, S3, label='Sentinel + High Friction ($\Omega=1.4$)', color='#d62728', linewidth=3)

# Threshold Definitions

plt.axhline(y=30, color='gray', linestyle='--', alpha=0.6)

plt.text(5, 32, 'Brain Fog Threshold (Critical $S$)', color='gray', fontsize=10, fontweight='bold')

# Insolvency Zone Shading (Shutdown)

plt.fill_between(time, 0, 30, color='red', alpha=0.08, label='Insolvency Zone (Shutdown)')

# Title and Labeling using LaTeX formatting

plt.title('Dynamic Solvency Integration (HEPOE Theory)\n$S(t) = S_0 + \int_{0}^{t} [\Phi - (H(E) + \Delta P + W_{erasure}) \cdot \Omega] dt$', fontsize=15, pad=20)

plt.xlabel('Time (minutes)', fontsize=12)

plt.ylabel('Solvency Level $S(t)$ (%)', fontsize=12)

plt.ylim(0, 110)

plt.grid(True, which='both', linestyle='--', alpha=0.4)

plt.legend(loc='upper right', frameon=True, shadow=True)

# Finalization

plt.tight_layout()

plt.savefig('hepoe_dynamic_solvency_validation_en.png', dpi=300)

# plt.show() # Uncomment to view locally

**Figure 11:**

import numpy as np

import matplotlib.pyplot as plt

import seaborn as sns

def generate_figure_10():

# Chart style to maintain consistency with other figures

sns.set_theme(style="whitegrid")

plt.style.use('default')

# Time Parameters (e.g., a standard 8-hour sleep/rest cycle)

time_hours = np.linspace(0, 8, 200)

# Initial condition: The system enters rest starting from the Brain Fog limit (30%)

S_init = 30

# Recovery Rates (Integral: eta * Phi - H_min)

# Fast Re-solver: High efficiency, recharges quickly

rate_fast = 18

# Slow Re-solver: High friction or noisy environment, recharges slowly

rate_slow = 6

# Solvency Calculation (capped at the 100% ceiling)

S_fast = np.minimum(100, S_init + rate_fast * time_hours)

S_slow = np.minimum(100, S_init + rate_slow * time_hours)

# Figure Configuration

plt.figure(figsize=(10, 6))

# Plotting Recovery Curves

plt.plot(time_hours, S_fast, color='#2ca02c', linewidth=3,

label=r'Fast Re-solvers ($\eta \cdot \Phi \gg 1$)')

plt.plot(time_hours, S_slow, color='#d62728', linewidth=3,

label=r'Slow Re-solvers ($\eta \cdot \Phi \approx 1$)')

# Baseline (Total Readiness)

plt.axhline(y=100, color='black', linestyle='--', linewidth=2,

label=r'Total Systemic Readiness ($S_0$)')

# Visually highlighting Residual Insolvency

# Draws a double arrow showing the gap between where the Slow Re-solver stopped and 100%

plt.annotate('', xy=(8, 100), xytext=(8, S_slow[-1]),

arrowprops=dict(arrowstyle='<->', color='black', lw=1.5))

plt.text(7.8, (100 + S_slow[-1])/2, 'Residual\nInsolvency\n(Thermal Debt)',

ha='right', va='center', fontsize=11, fontweight='bold', color='black')

# Marking when the Fast Re-solver reaches 100%

t_full_fast = (100 - S_init) / rate_fast

plt.plot(t_full_fast, 100, marker='o', markersize=8, color='#2ca02c')

# Texts, Titles, and Formatting

plt.title('Recovery Kinetics and Residual Insolvency', fontsize=14, fontweight='bold', pad=15)

plt.xlabel('Rest / Sensory Isolation Time (hours)', fontsize=12)

plt.ylabel('Solvency Capacity (%)', fontsize=12)

# Axis limits

plt.ylim(0, 110)

plt.xlim(0, 8.5)

# Legend and Grid

plt.legend(loc='lower right', frameon=True, shadow=True, fontsize=11)

plt.grid(True, linestyle='--', alpha=0.5)

plt.tight_layout()

# Save the image in high resolution

plt.savefig('Recovery_Kinetics.png', dpi=300)

plt.show()

# Execute the function

if __name__ == "__main__":

generate_figure_10()
