# Supplementary material for: The Sentinel Phenotype: a theoretical bioenergetic and neurobiological framework for high-fidelity predictive systems (HEPOE Theory)
Source: Front Neurosci. 2026 May 29;20:1785088. doi: 10.3389/fnins.2026.1785088 (PMC13260446; doi:10.3389/fnins.2026.1785088)
Supplement: Supplementary file 2 [file Data_Sheet_2.docx]

**DATA SHEET 2: SYSTEMIC FRICTION COEFFICIENT (Ω)
AND BIOLOGICAL SCALING**

| **Condition / Biomarker** | **Friction / Impedance Level (Ω)** | **Impact on Solvency (*S*(*t*))** | **Biophysical Justification** |
| --- | --- | --- | --- |
| **Optimized Hardware** | 1.0 | Sustainable long-term cognitive activity. | Baseline metabolic restoration flux matches high-fidelity load. |
| **Mild Inflammation / Nutritional Deficit** | 1.1 - 1.2 | Accelerated solvency decay. | Increased oxidative stress reduces ATP efficiency. |
| **Enzymatic Deficiencies / Chronic Inflammation / Prolonged “Masking”** | 1.3 - 1.5 | Rapid Insolvency. | High systemic friction and structural impedance leads to the HEPOE Breakpoint up to 50% faster. Genetic polymorphisms impacting mitochondrial ATP yield or chronic immune activation. |
| **Severe Allostatic Load / Neurotoxicity** | > 1.5 | Immediate Collapse. | System enters the Insolvency Zone (*S* < 30%) shortly after task initiation. |

Source: Brezolin and Freitas (2026).
